# Structural basis of host protein hijacking in human T-cell leukemia virus integration

**DOI:** 10.1038/s41467-020-16963-6

**Published:** 2020-06-19

**Authors:** Veer Bhatt, Ke Shi, Daniel J. Salamango, Nicholas H. Moeller, Krishan K. Pandey, Sibes Bera, Heather O. Bohl, Fredy Kurniawan, Kayo Orellana, Wei Zhang, Duane P. Grandgenett, Reuben S. Harris, Anna C. Sundborger-Lunna, Hideki Aihara

**Affiliations:** 1 The Hormel Institute, University of Minnesota, 801 16th Avenue N.E., Austin, MN 55912 USA; 2 Masonic Cancer Center, University of Minnesota, 2231 6th Street S.E., Minneapolis, MN 55455 USA; 3 Department of Biochemistry, Molecular Biology and Biophysics, University of Minnesota, 321 Church Street S.E., Minneapolis, MN 55455 USA; 4 Institute for Molecular Virology, University of Minnesota, 515 Delaware Street S.E., Minneapolis, MN 55455 USA; 5 Department of Molecular Microbiology and Immunology, Saint Louis University, 1100 S. Grand Boulevard, St. Louis, MO 63104 USA; 6 Department of Diagnostic and Biological Sciences, School of Dentistry, University of Minnesota, 515 Delaware Street S.E., Minneapolis, MN 55455 USA; 7 Characterization Facility, College of Science and Engineering, University of Minnesota, 100 Union Street S.E., Minneapolis, MN 55455 USA; 8 Howard Hughes Medical Institute, University of Minnesota, 2231 6th Street S.E., Minneapolis, MN 55455 USA

**Keywords:** Retrovirus, Cryoelectron microscopy

## Abstract

Integration of the reverse-transcribed viral DNA into host chromosomes is a critical step in the life-cycle of retroviruses, including an oncogenic delta(δ)-retrovirus human T-cell leukemia virus type-1 (HTLV-1). Retroviral integrase forms a higher order nucleoprotein assembly (intasome) to catalyze the integration reaction, in which the roles of host factors remain poorly understood. Here, we use cryo-electron microscopy to visualize the HTLV-1 intasome at 3.7-Å resolution. The structure together with functional analyses reveal that the B56γ (B’γ) subunit of an essential host enzyme, protein phosphatase 2 A (PP2A), is repurposed as an integral component of the intasome to mediate HTLV-1 integration. Our studies reveal a key host-virus interaction underlying the replication of an important human pathogen and highlight divergent integration strategies of retroviruses.

## Abstract (web summary)

Integration of the reverse-transcribed viral DNA into host chromosomes is a critical step in the life-cycle of retroviruses such as the human T-cell leukemia virus type-1 (HTLV-1). Here the authors describe the structural role of the host co-factor protein phosphatase 2A B56γ subunit in supporting integration as part of the HTLV-1 intasome.

## Introduction

Over 10 million people worldwide are infected with human T-cell leukemia virus type-1 (HTLV-1), an oncogenic delta(δ)-retrovirus related to HIV-1^1,2^. HTLV-1 infection causes an aggressive CD4^+^ T-cell malignancy known as adult T-cell leukemia/lymphoma (ATL) after a latency period up to several decades^3,4^. HTLV-1 infection can also cause a chronic inflammatory disease of the spinal cord known as HTLV-1-associated myelopathy/tropical spastic paraparesis (HAM/TSP)^5,6^. However, despite the importance of HTLV-1 as the causative agent of these diseases, there is no therapeutic intervention against HTLV-1 infection or its diseases. A critical step and the hallmark of retroviral infection is the integration of a reverse-transcribed viral genome into host chromosomal DNA^7^. Integration is carried out by the virally encoded integrase (IN) enzyme, which forms a higher-order nucleoprotein assembly (intasome) and catalyzes the 3′-end resection of a linear reverse-transcribed viral DNA and the subsequent direct attack on a target cellular DNA backbone by the nascent viral DNA 3′-OH termini^8–10^. Due to its essential role in the viral lifecycle, HIV-1 IN is the target of several clinically used antiviral drugs (INSTIs: IN strand-transfer inhibitors) that selectively inhibit the latter strand-transfer step and also inhibit INs from other retroviruses, including HTLV-1^7,11–13^.

Structural studies have revealed a remarkable diversity of retroviral IN–DNA complex assemblies, ranging from tetrameric IN for a spumavirus prototype foamy virus (PFV)^12,14^, octameric IN for an alpha-retrovirus Rous sarcoma virus (RSV)^15,16^ and a beta-retrovirus mouse mammary tumor virus (MMTV)^17^, to even hexadecameric IN for a lentivirus maedi-visna virus (MVV)^18^ (Supplementary Fig. 1a, c, d). IN from another lentivirus, and an important retroviral human pathogen, HIV-1, has been reported to form a heterogeneous mixture of tetrameric to dodecameric complexes^19^. In addition to this structural diversity, INs from different genera of retroviruses bind to distinct host co-factors^20–23^, and allosteric IN inhibitors (ALLINs) that target the HIV-1 IN-LEDGF/p75 interface are being developed as novel antivirals for their capacity to modulate IN multimerization and inhibit late replication steps^24–27^. Recent studies have identified a host serine/threonine phosphatase PP2A comprising the B56 regulatory subunit as the functional binding partner for IN from deltaretroviruses including HTLV-1^28^. However, structural information is lacking for a deltaretroviral intasome, and it is unknown how the unique co-factor PP2A stimulates the concerted integration activity or regulates the integration-site selection of deltaretroviral INs. In this study, we use cryo-electron microscopy (cryo-EM), virus infectivity assays, and biochemical analyses to show that PP2A-B56γ is an integral component of the HTLV-1 intasome that plays an important role in HTLV-1 infection.

## Results

### Structure determination of the HTLV-1 intasome

**Fig. 1:**
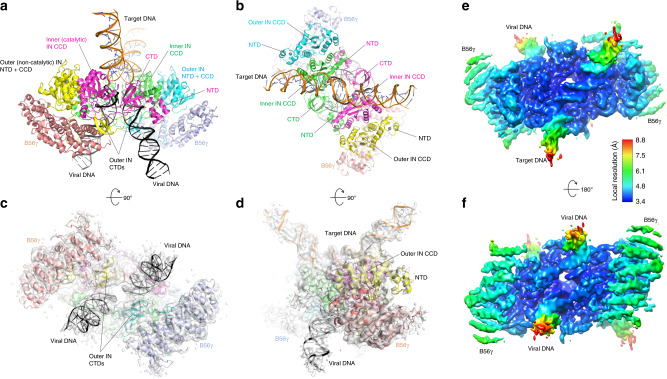
Overall structure of the HTLV-1 intasome. **a** A view along a direction perpendicular to the overall twofold symmetry axis of the complex. Each protein chain is colored differently. Viral and target DNA are shown as black and orange ribbons, respectively. Zinc ions are shown as gray spheres. **b** A view along the twofold axis, from the target DNA side. **c**, **d** Views after 90° rotation of **a** and **b**, respectively, with the cryo-EM map in transparent surface overlaid on the atomic models. **e, f** Cryo-EM map in solid surfaces colored according to local resolution.

To address the knowledge gap described above, we determined the structure of the HTLV-1 intasome using cryo-EM and single particle analysis at 3.7-Å resolution (Supplementary Figs. 2 and 3; Table 1). We assembled a stable complex including HTLV-1 IN, a fragment of human B56γ spanning residues 11–380^29^, and a branched DNA molecule containing the viral U5 long terminal repeat (LTR) sequence^30,31^ and a target DNA, mimicking the product of the concerted strand-transfer reactions. We found the presence of B56γ to be essential for a stable HTLV-1 IN–DNA complex formation, consistent with its reported strong stimulatory effect on deltaretroviral concerted integration reactions under certain biochemical conditions^11,28^. SDS-PAGE analysis confirmed that B56γ(11–380) is part of the size-exclusion chromatography (SEC)-isolated HTLV-1 intasome. The molecular mass of the HTLV-1 intasome, or the strand-transfer complex (STC) thus formed, was estimated to be 345 and 305 kDa in solution by SEC-coupled multiangle light scattering (SEC-MALS) and mass photometry analyses, respectively (Supplementary Fig. 2). In accordance with these observations, the cryo-EM density map shows a complex with the total mass of 320 kDa, including an IN tetramer bound to a strand-transfer product DNA and two molecules of B56γ (Fig. 1).

**Table 1:** Cryo-EM data collection, refinement, and validation statistics.

	HTLV-1 intasome (EMDB-21301) (PDB: 6VOY)
*Data collection and processing*	
Magnification	96,000×
Voltage (kV)	300
Electron exposure (e^–^/Å^2^)	30
Defocus range (μm)	1.0–2.0
Pixel size (Å)	0.8933
Symmetry imposed	C2
Initial particle images (no.)	1,184,769
Final particle images (no.)	30,434
Map resolution (Å)	3.7
FSC threshold	0.143
Map resolution range (Å)	3.4–7.1
*Refinement*	
Initial model used (PDB code)	5EJK
Model resolution (Å)	3.7
FSC threshold	0.143
Map sharpening *B* factor (Å^2^)	−30
Model composition	
Non-hydrogen atoms	17,558
Protein residues	1724
DNA residues	180
Ligands (Zn^2+^, Mg^2+^)	4,2
*B* factors (Å^2^)	
Protein	181.09
DNA	300.68
Ligand	203.04
R.m.s. deviations	
Bond lengths (Å)	0.010
Bond angles (°)	1.131
Validation	
MolProbity score	2.26
Clashscore	33.53
Poor rotamers (%)	0.66
Ramachandran plot	
Favored (%)	96.29
Allowed (%)	3.71
Disallowed (%)	0.00

### IN tetramer in the HTLV-1 intasome

**Fig. 2:**
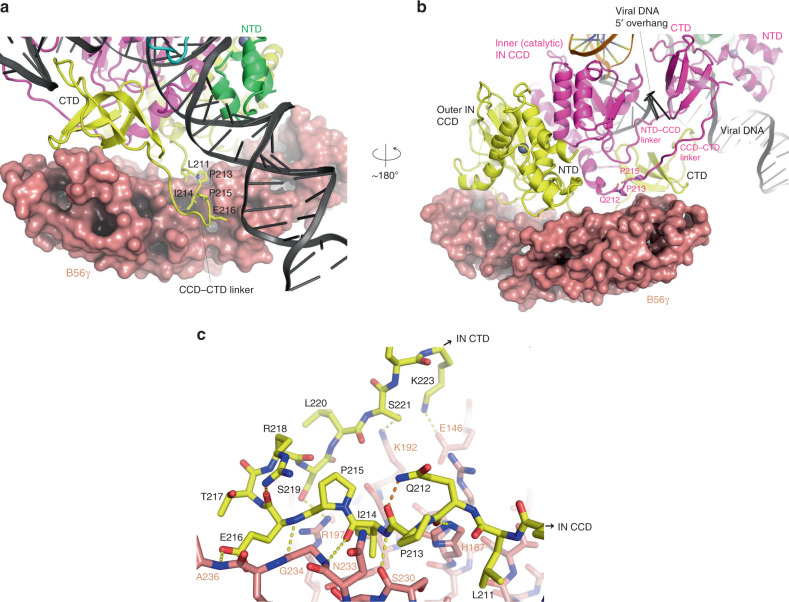
B56γ–IN interface. **a** A close-up view centered on the CCD–CTD linker of outer non-catalytic IN (yellow) containing the ^211^**L**QP**I**P**E**^216^ short linear motif, which is docked in the central cleft of B56γ. Molecular surface is shown for B56γ. **b** A view from the opposite side of B56γ. The CCD–CTD linker of inner catalytic IN (magenta) traverses the B56γ surface. **c** A network of hydrogen-bonds and salt-bridges mediating the binding of IN CCD–CTD linker in a U-shaped conformation to B56γ. Intermolecular and intramolecular contacts are highlighted by yellow and orange dotted lines, respectively.

HTLV-1 intasome has a twofold symmetric structure that shares the conserved intasome core (CIC)^10^ with the intasome assemblies of other genera of retroviruses. The IN tetramer consists of two inner catalytic and two outer non-catalytic subunits. Each inner IN comprises reciprocally swapped N-terminal domain (NTD) bound over the viral DNA major groove, a NTD-CCD linker that contacts both viral DNA ends, the catalytic core domain (CCD) engaging the viral/target DNA junction in the minor groove, and the C-terminal domain (CTD) that fits between the NTD and CCD of the same molecule in *cis* (Fig. 1b; Supplementary Figs. 4b and 5a–c). Both NTD-CCD and CCD–CTD linkers of the inner catalytic IN run across the synaptic interface, arranged antiparallel to each other and interacting with the 5′ overhang of viral DNA non-transferred strand (Fig. 2b). CTD of the non-catalytic outer INs further contribute to the extensive viral DNA interaction, bridging between the two viral DNAs attached to opposing strands of the target DNA (Fig. 1a, c, f, Supplementary Figure 5e). The target DNA shows a kink at each of the viral/target DNA junctions 6 bp apart, resulting in a total bending of ~80° away from the intasome core (Fig. 1d). The configuration of viral and target DNA molecules is similar to that observed in RSV intasome^15^, which shares a 6-bp spacing between the strand-transfer points. This similarity includes a zigzagged trajectory of the target DNA with an offset of the helical axes in the direction perpendicular to that of the overall bending (Fig. 1b, e; Supplementary Fig. 1).

### PP2A-B56γ–IN interaction

Two molecules of the deltaretrovirus-specific host co-factor B56γ are bound symmetrically to the core of the HTLV-1 intasome flanking the viral DNAs, as though to cradle the IN tetramer (Fig. 1a, c). Both inner and outer subunits of an IN dimer on each side of the intasome fit in the concave surface of B56γ (Fig. 2; Supplementary Fig. 5d). CCD and CTD of the outer non-catalytic IN are bound toward either end of the banana boat-shaped B56γ monomer^29^ (Fig. 2b), while the inter-domain linker between CCD and CTD takes a U-shaped conformation and makes an anchoring interaction in the central peptide-binding cleft of B56γ (Fig. 2a, c). The ^211^**L**QP**I**P**E**^216^ sequence from the CCD–CTD linker, previously shown to be critical for the binding of HTLV-1 IN to PP2A-B56^32^, docks into a highly conserved binding pocket known to bind the “LxxIxE” short linear motif found in a number of host proteins regulated by PP2A^32–34^. Residues after the sharp U-turn, ^219^SLSNK^223^, interact with charged amino acids on the B56γ surface, including Arg197 (Fig. 2c; Supplementary Fig. 6). The CCD–CTD linker of the inner catalytic IN also traverses across the B56γ surface, running normal to the axes of the pseudo-HEAT repeat α-helices (Fig. 2b). Consistent with the observed mode of interaction between B56γ and IN, we found that the CCD–CTD 2-domain fragment of HTLV-1 is necessary and sufficient for forming a stable complex with B56γ isolable by SEC, and this interaction is abolished by mutating ^211^**L**QP**I**P**E**^216^ to ^211^**A**QP**A**P**A**^216^ (Supplementary Fig. 7). B56γ appears to stabilize each IN dimer, help organize the CCD–CTD linkers, and position CTDs for viral DNA interactions. The distinct conformations of the HTLV-1 IN CCD–CTD linkers mediating B56γ interaction contrast those of the much longer CCD–CTD linker of PFV IN^12,14^, extended conformations of the short CCD–CTD linkers of RSV IN^15,35,36^ and MMTV IN^17^, and a crossed α-helical bundle structure assumed by the lentiviral IN CCD–CTD linkers^18,19,37^.

### B56γ is important for HTLV-1 integration in cells

**Fig. 3:**
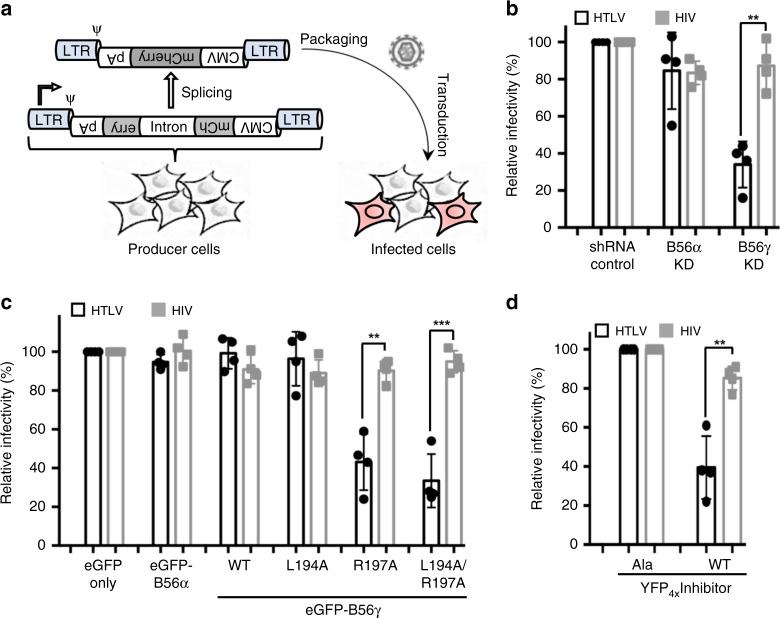
B56γ plays an important role in HTLV-1 infection. **a** A schematic diagram of the HTLV-1 and HIV-1 infection assay system. Fluorescence signal can only be generated following removal of the intron disrupting *mCherry* (producer cells) and subsequent reverse-transcription and integration of the viral genome (infected cells). **b** Relative infectivity of the indicated retroviral vectors in *B56α* or *B56γ* knockdown cells compared to controls. **c** Relative infectivity of the indicated retroviral vectors in cells overexpressing the indicated B56 construct compared to eGFP alone. **d** Relative infectivity of the indicated retroviral vectors in cells overexpressing four copies of a repeat peptide sequence containing **L**PT**I**H**E** (WT) compared to cells expressing four copies of the peptide sequence **A**PT**A**H**A** (Ala). **P < 0.01; ***P < 0.001 by the unpaired Student *t* test. Data shown are from four independent replicates.

Our structural data suggest that B56γ, which is a constitutively nuclear member of the PP2A B-subunit family, may play a key role in HTLV-1 integration as a scaffolding component or a regulator of the intasome assembly. To test whether B56γ is required for HTLV-1 integration in human cells, we performed HTLV-1 infectivity assays in the presence or absence of B56γ or the closely related cytoplasmic family member B56α (75% identity and 88% similarity within the core domain). As a control, HIV-1 infectivity was assessed in parallel to determine the requirement for B56γ in general retroviral genome integration. To perform HTLV-1 and HIV-1 infectivity assays, we used reverse-intron containing reporter vectors that only generate fluorescence upon successful integration of the viral genome into target cells^38^ (Fig. 3a). Based on our structural data and subcellular localization, we predicted that shRNA-mediated knockdown of *B56γ*, but not *B56α*, would significantly impair HTLV-1 infectivity while HIV-1 infectivity would remain unchanged. As we predicted, HIV-1 infectivity showed no differences in control or knockdown cells; however, HTLV-1 infectivity was significantly impaired in cells stably expressing shRNA against *B56γ*, but not *B56α* (Fig. 3b). Of note, efficient and selective depletion of the targeted transcript in these shRNA-expressing cell lines was confirmed previously, and it was shown that neither *B56γ* nor *B56α* knockdown has discernable effect on the cell cycle^39^.

**Fig. 4:**
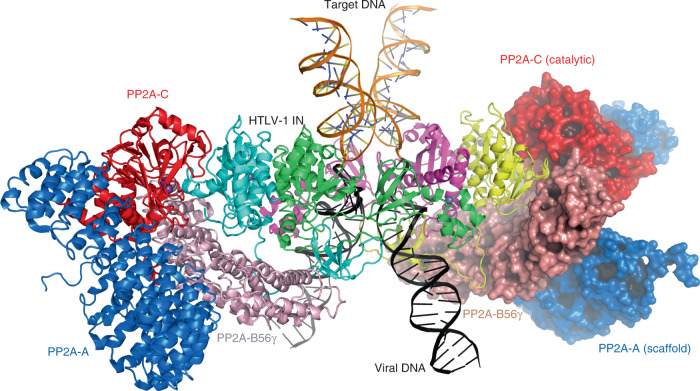
A hypothetical model of HTLV-1 intasome containing PP2A holoenzymes. Two copies of PP2A holoenzyme^50^ were docked on the HTLV-1 intasome by superposition of the B56γ subunits, resulting in a snug fit between the catalytic C subunit of PP2A (red) and the catalytic domain of outer IN subunit (yellow and cyan).

To independently probe the requirement for B56γ in HTLV-1 integration, we assessed infectivity in cells overexpressing either wild-type B56γ or mutant variants that we predicted to have compromised interaction with HTLV-IN. As expected, all cell lines overexpressing the indicated B56 variants had a minimal impact on HIV-1 infectivity (Fig. 3c). Interestingly, cells expressing either B56γ R197A or L194A/R197A had a significant impact on HTLV-1 infectivity, while the L194A variant alone had no effect (Fig. 3c). These findings are consistent with our structural data (Fig. 2c; Supplementary Fig. 6) and previous biochemical observations^28^ that indicated that Arg197 plays a more important role in HTLV-IN binding to B56γ than Leu194. The dominant-negative effect of overexpressing the R197A variant of B56γ could be because of its residual interaction with IN and resulting interference with intasome formation. An alternative scenario is that HTLV-1 integration in cells actually requires the heterotrimeric PP2A holoenzyme containing B56γ (see “Discussion” below and Fig. 4) and that overexpressing the defective B56γ depleted the pool of PP2A holoenzyme containing the wild-type B56γ capable of supporting IN function.

We further examined the impact of B56γ on HTLV-1 integration by using a previously characterized high-affinity peptide inhibitor that has been shown to suppress Ebola virus infection by competitively inhibiting the nucleoprotein binding to B56 proteins^40^. Co-expression of a plasmid containing four copies of the LxxIxE peptide motif with HTLV-1 producing plasmids resulted in a significant decrease in HTLV-1 infectivity, while HIV-1 infectivity only displayed a modest decrease (Fig. 3d). However, when four copies of the control inhibitor (AxxAxA) were expressed, we observed no discernable effect on HTLV-1 or HIV-1 infectivity. Taken together, these results support our structural data that B56γ is an important component of the HTLV-1 intasome.

## Discussion

The observed mode of interaction with IN by PP2A-B56γ is distinct from those previously seen for the cellular co-factors of retroviral IN from other genera, LEDGF/p75 for lentivirus^41,42^ and the BET family proteins for a gammaretrovirus murine leukemia virus (MLV)^43^, both of which stimulate the concerted integration activity and dictate the unique integration-site preferences of cognate IN^20,24,44–46^. The divergent IN–host factor interactions parallel the diverse strategies employed by these INs, including their different domain arrangements and oligomeric structures^10^, in achieving the stable CIC structure important for catalysis. A unique solution by deltaretroviruses is to hijack and re-purpose the nuclear localized subunit of a host enzyme PP2A for stabilizing the intasome assembly (Supplementary Fig. 1). PP2A is a major regulator of cell cycle and involved in numerous cellular signaling pathways, and as such, it is targeted or hijacked by a number of viruses including HIV-1^39,40,47,48^. Notably, HTLV-1 trans-activator protein Tax has been reported to inhibit PP2A catalytic activity to manipulate cellular signaling, thereby achieving constitutive activation of NF-κB^49^. It remains to be further investigated whether the B56-IN interaction has any outcome that involves the PP2A phosphatase activity. Regardless, a simple modeling exercise suggests that PP2A holoenzyme, complete with all three subunits^50,51^, can fit in the HTLV-1 intasome without significant steric clashes (Fig. 4), consistent with the reported association of PP2A holoenzyme comprising the B56 subunits with deltaretroviral IN^28^. Thus, it is possible that the large scaffold (A) or the catalytic (C) subunit of the PP2A holoenzyme plays a role in deltaretroviral integration either through its catalytic activity or mediating additional protein interactions. As previously suggested, PP2A may direct HTLV-1 integration into transcriptionally active regions via its interaction with certain chromatin-associated proteins^28,52,53^. The HTLV-1 intasome structure reported here provides mechanistic insights into a critical host-pathogen interaction underlying the replication of an important human pathogen and affords a framework for the future development of novel therapeutic interventions against HTLV-1 infection or replication.

## Methods

### Protein purification

HTLV-1 IN suffers from poor solubility and is prone to aggregation, making structural studies difficult. To overcome this problem, we adapted the Sso7d-fusion strategy previously used successfully in the structural studies of HIV-1 IN^19^. Full-length HTLV-1 IN was expressed in *E. coli* strain BL21(DE3) with a 6xHis-tag and a DNA-binding defective mutant of Sso7d (W24A/R43E) fused to its N-terminus and purified using nickel-affinity and gel-filtration chromatography. Purified Sso7d-IN in 20 mM HEPES-NaOH (pH 7.5), 1.0 M NaCl, 5% glycerol, 0.5 mM TCEP, was concentrated to ~20 mg ml^−1^ by ultrafiltration. Sso7d (W24A/R43E)-HTLV-1 IN(wt) exhibited robust concerted integration activity, which was modestly stimulated by B56γ and inhibited by dolutegravir (Supplementary Fig. 8) and showed the expected 6-bp spacing between the integration sites on opposing DNA strands (Supplementary Fig. 9). For the intasome assembly, we used a catalytically inactive E156Q mutant of HTLV-1 IN. B56γ(11–380)^29^ was expressed in *E. coli* BL21(DE3) as a 6xHis-Sumo-fusion protein and purified as above. For intasome assembly (Supplementary Fig. 2), the N-terminal 6xHis-Sumo tag was removed by Ulp1 protease treatment during purification. Purified B56γ(11–380) in 20 mM Tris-HCl pH 7.4, 0.5 M NaCl, 5 mM β-mercaptoethanol was concentrated to ~40 mg ml^-1^. For in vitro protein–protein-binding studies (Supplementary Fig. 7), various HTLV-1 IN fragments (NTD-CCD, CCD, CCD–CTD, CTD) and B56γ(11–380) were expressed and purified as Sumo-fusion proteins and used without removing the 6xHis-Sumo tag. The amino acid sequences of the protein constructs used in this study are shown in Supplementary Table 1. All purified proteins were flash-frozen in liquid nitrogen and stored at −80 °C until use.

### HTLV-1 intasome preparation

A mixture containing 60 μM each of Sso7d-IN(E156Q) and B56γ(11–380), 50 μM each of the three pre-annealed oligonucleotides (U5-25T20, U5-nj25, Target20), 20 mM HEPES-NaOH (pH 7.5), 1.0 M NaCl, 10 mM DTT, 25% glycerol, and 10 mM MgCl_2_ was dialyzed at room temperature for ~16 h against 45 mM Tris base, 45 mM boric acid, 0.1 M NaCl, 50 mM MgSO_4_, 25% glycerol, and 0.5 mM TCEP. The mixture after dialysis was supplemented with 150 mM NaCl and left at room temperature for ~30 min, which helped to re-solubilize some of the precipitated IN–DNA complex. Following a brief centrifugation to remove insoluble aggregates, the protein/DNA mixture was either flash-frozen and stored for SEC-MALS analyses or injected into a Superdex200 Increase 10/300 SEC column equilibrated with the SEC buffer consisting of 20 mM Tris-HCl (pH 8.0), 0.5 M NaCl, 1 mM MgCl_2_, and 0.5 mM TCEP, operating at room temperature. The IN-B56γ-DNA complex peak (Supplementary Fig. 2a, b) was used for EM grid preparation or mass photometry analysis. No IN–DNA complex was formed in the absence of B56γ(11–380).

### Cryo-EM imaging and data processing

A 3.5 µL aliquot of SEC-purified HTLV-1 intasome was applied to Quantifoil R1.2/1.3 grids (Electron Microscopy Sciences) and vitrified in liquid ethane using a Mark IV Vitrobot (Thermo Fisher). Grids were imaged in a 300 kV Titan Krios electron microscope, and a total of 3326 micrographs (image stacks) were acquired with a Falcon III direct electron detector using EPU (Thermo Fisher) at a nominal magnification of 96,000×, corresponding to 0.89 Å/pixel. Motion correction was carried out using MotionCor2^54^ on dose-weighted images, after deleting the first two frames. Contrast transfer function (CTF) estimation was carried out using Gctf^55^ without dose weighting. All subsequent data processing and refinement steps were carried out in RELION3^56^ (Supplementary Fig. 3). A small subset of micrographs was used for reference-free automated particle picking using a Laplacian of Gaussian filter, which generated a pool of 68,996 particles. 2D classification of this data set generated templates for automated particle picking from all micrographs and the resulting 1,184,769 particles were downscaled to 3.54 Å/pixel, and subjected to several iterative rounds of 2D classification to obtain a final pool of 181,688 good particles. These refined particles were re-extracted at the original pixel size of 0.89 Å/pixel, subjected to several rounds of iterative 3D classification and 3D-refinement. To improve map quality, C2 symmetry was imposed during subsequent rounds of 3D classification and refinement. Combining the half-data sets and flattening the solvent density resulted in a map of 4.1-Å resolution. The mask used to flatten the solvent density was generated in RELION3^56^ at a threshold where one of the low-pass filtered half-maps stopped displaying any noise outside the reconstruction using Chimera to display the map^57^. CTF refinement was employed to estimate per-particle defocus values and to refine beam tilt values. Beam-induced motion was corrected using Bayesian methods. Polished particles were subjected to one round of 3D classification leading to a final pool of 30,434 particles and a final round of 3D-refinement was carried out using a solvent mask and enabling phase-randomization based correction of Fourier shell correlation (FSC)^58^ curves in every iteration of the refinement. The resolution of the final map was estimated to be 3.7 Å using the “gold standard” (FSC = 0.143)^59^. Directional FSC plot (Supplementary Fig. 3l) was generated using 3DFSC^60^. Local variation in resolution was estimated using RELION3^56^ (Fig. 1e, f).

### Model building and refinement

Homology models of HTLV-1 IN structural domains were generated using MODELLER^61^ and Phyre2^62^ based on the high-resolution crystal structures of RSV and HIV-1 IN domains^35–37,63^ and placed into the cryo-EM map. The linker segments and DNA molecules were built manually in COOT^64^. B56γ crystal structure^29^ was docked as a rigid body into the map. The preliminary model thus obtained was refined using PHENIX^65^ real_space_refine against the cryo-EM density and a standard set of geometry/stereochemistry restraints. The resulting model closely matched an unbiased model generated de novo by PHENIX autobuild function, with the RMSD of protein main chain atoms of ~0.9 Å. A summary of the cryo-EM data collection/processing and model refinement statistics is shown in Table 1. Molecular graphics images were generated using PyMOL (www.pymol.org) or UCSF Chimera^57^.

### Size exclusion and light scattering

The SEC-MALS data were collected using a Superdex200 10/300 HR SEC column (GE Healthcare), connected to Agilent 1200 high performance liquid chromatography (HPLC) system, equipped with an autosampler. The elution from SEC was monitored by a photodiode array (PDA) UV/VIS detector (Agilent Technologies), differential refractometer (OPTI-Lab rEx Wyatt Technology), static and dynamic, multiangle laser light-scattering (LS) detector (HELEOS II with QELS capability, Wyatt Technology). The SEC-UV/LS/RI system was equilibrated with 20 mM Tris-HCl (pH 8.0), 0.5 M NaCl, 1 mM MgCl_2_, and 1 mM DTT at the flow rate of 0.5 ml min^−1^. Two software packages were used for data collection and analysis; the Chemstation software (Agilent Technologies) controlled the HPLC operation and data collection from the multi-wavelength UV/VIS detector, while the ASTRA software (Wyatt Technology) collected data from the refractive index (RI) detector, the light-scattering detectors, and recorded the UV trace at 280 nm sent from the PDA detector. The weight average molar masses, *Mw*, were determined across the entire elution profile in the intervals of 2 s from static LS measurement using ASTRA software as previously described^66,67^. During data analysis, a dn/dc value of 0.188 mL g^−1^ was used as it proved satisfactory during analyses of protein standards analyzed before and after the samples of interest. The IN-B56γ-DNA complex eluted with a *Mw* of ~ 345 kDa; there were no changes in *Mw* when the complex was analyzed at a fivefold lower concentration (Supplementary Fig. 2c). Additional information about the stoichiometry of the protein–DNA complex was obtained from the UV/RI ratio, which is proportional to extinction coefficient of the molecule, measured individually for the proteins alone, DNA alone, and the complex samples. Since the UV/RI ratio for DNA was 7.5 times higher than the value observed for proteins, the parameter is very sensitive to the protein to DNA ratio present in the eluting complex. The observed UV/RI ratio for the eluting complex closely matched, with 1% deviation, the value calculated for the stoichiometry observed in the cryo-EM structure.

### Plasmids and cloning for cell-based assays

The eGFP control and B56 expression vectors used in this study were cloned into the pQCXIH retroviral expression vector as described previously^39^. B56γ point mutants were generated by PCR amplification using Phusion high-fidelity DNA polymerase (NEB, Ipswich, MA) and overlapping PCR to introduce the indicated mutations. To generate the wild-type and alanine YFP_4×_Inhibitor vectors, cDNA sequences were obtained as gBlocks from Integrated DNA Technologies (IDT) and cloned into pcDNA5TO expression vectors using *Hind*III and *Not*I restriction enzymes. The sequences used for generating the inhibitor vectors have been described previously^40^. The control and knockdown shRNA constructs have also been described previously^39^. All constructs were confirmed by restriction digestion and Sanger sequencing.

### Cell lines and culture conditions

293T HEK cells were maintained in DMEM (Hyclone, South Logan, UT) supplemented with 10% FBS (Gibco, Gaithersburg, MD) and 0.5% pen/strep (50 units). 293T cells were transfected with TransIT LTI (Mirus, Madison, WI) according to the manufacturer’s protocol. To generate stable eGFP, B56α, and B56γ wild-type and mutant cell lines, viruses were produced from 293T cells transfected with the pQCXIH retroviral expression vectors described above, an MLV GagPol packaging vector, and a VSV-G expression vector. Media was harvested 48 h post transfection and frozen at −80 °C for 4–6 h, thawed and centrifuged at 1500×*g*, and combined with fresh 293T cells. To generate pure cell populations, samples were treated with hygromycin B (Sigma, 200 μg/ml) 48 h post transduction. For generating stable shRNA knockdown/vector control lines, 293T cells were transfected with the shRNA vector, an HIV-1 Gag/Pol packaging construct, and a VSV-G expression vector. Media was harvested 48 h post transfection, and frozen at −80 °C for 4–6 h, thawed and centrifuged at 1500×*g*, and combined with fresh 293T cells. Pure cell populations were generated by treating with puromycin for 48 h to produce a pure population (Sigma, 1 μg/ml).

### HIV-1 and HTLV-1 infectivity assays

A one-step transfection/infection assay was performed in 293T cells using a 12-well culture plate. Roughly, 150,000 cells (either non-transduced 293T cells, cells stably expressing the indicated eGFP-B56 proteins, or cells stably expressing the indicated shRNA vector), were seeded into 12-well plates and allowed to adhere overnight. After 24 h, cells were transfected with either 0.6 μg of an HIV-1 packaging vector, 0.15 μg of VSV-G expression vector, and 0.9 μg of reporter plasmid, or, 0.6 μg of the full-length HTLV-1 pCMV HT1-M genome, and 0.9 μg of a reporter plasmid. The generation of these vectors has been described previously^38^. To test the YFP_4×_Inhibitor vectors, the aforementioned HIV/HTLV transfection protocols were followed along with 0.4 μg of either wild-type or alanine inhibitor plasmids. Media was changed 16 h following transfection, and the cells were harvested 5 days later, and infectivity was assessed using flow cytometry (representative gating approaches are depicted in Supplementary Fig. 10). The data were collected from four independent experiments, and infectivity results were plotted using GraphPad Prism 6 software with error bars representing standard error mean (SEM).

### Mass photometry

Microscope coverslips (High Precision coverslips, No. 1.5, 24 × 50 mm, ThorLabs) were cleaned by sequential washing in 100% isopropanol and Milli-Q H_2_O, followed by drying with a clean air stream. Silicon gaskets (Grace bio-labs, CultureWell™ Reusable Gaskets, CW-50R-1.0) were placed on the clean coverslips to create wells. Immediately prior to mass photometry measurements, protein or protein–DNA complex samples were diluted directly on the coverslip in the SEC buffer. Each sample was measured in a new well (i.e., each well was used once). To find focus, fresh buffer was first added into the well, the focal position was identified and secured in place with an autofocus system based on total internal reflection for the entire measurement. For each acquisition, 18–19 µL of buffer was introduced into the well and, following autofocus stabilization, 2–1 μL of sample was added then movies of 60-s duration were recorded. Data were acquired using a One^MP^ mass photometer (Refeyn Ltd, Oxford, UK). Data acquisition was performed using Acquire^MP^ (Refeyn LTD, v2.0) using default settings. Mass photometry movies were processed and analyzed using Discover^MP^ (v1.2.4) using default settings.

### In vitro integration assay

The concerted integration activity of Sso7d(W24A/R43E)-HTLV-1 IN(wt) was tested using a 3′-OH recessed viral DNA substrate 25 R containing the HTLV-1 U5 LTR sequence, prepared by annealing two HPLC-purified oligonucleotides 5′-Cy5-CCAGGAGAGAAATTTAGTACACA-3′ and 5′-ACTGTGTACTAAATTTCTCTCCTGG-3’ (IDT). The reaction mix initially included 0.5 μM viral DNA substrate and 1.5 μM IN in 25 mM HEPES (pH 7.0), 100 mM NaCl, 10 mM MgCl_2_, 10 µM ZnCl_2_, 10 mM dithiothreitol (DTT), and 10% (v/v) dimethyl sulfoxide (DMSO). After an initial preincubation at 14 °C for 15 min, the supercoiled target DNA, pBSKZeo (2.7 kb)^46^, was added to a final concentration of 8 nM, and strand transfer was carried out at 37 °C for 45 min. The reactions were stopped by adding EDTA to a final concentration of 25 mM, and samples were deproteinized with 0.5% SDS, 1 mg/ml proteinase K for 1 hr at 37 °C. Strand-transfer products were separated on a 1.5% agarose gel and visualized by scanning for Cy5 fluorescence on Typhoon 9500 Laser Scanner (GE Healthcare Life Sciences). The gel was stained with SYBR Gold (Invitrogen) and analyzed by a Typhoon 9500 scanner to visualize the target DNA (shown on the left and right, respectively, in Supplementary Fig. 8).

### HTLV-1 integration-site sequencing

The viral DNA–target junctions of the concerted integration products generated in vitro by Sso7d(W24A/R43E)-HTLV-1 IN(wt) were sequenced. The strand-transfer reactions were carried out as above, except with HTLV-1 U5 LTR DNA (39 CatRE: 5′- CCGTGCGAATTCGGATCCAGGAGAGAAATTTAGTACACA-3′ and 41 Non-CatRE: 5′- ACTGTGTACTAAATTTCTCTCCTGGATCCGAATTCGCACGG-3′) for 20 min at 37 °C. The concerted products were isolated from a 0.8% agarose gel and purified by electroelution. The products were treated with phi29 DNA polymerase (New England Biolabs) in the presence of 500 μM dNTPs followed by Klenow polymerase (Promega) treatment. The blunt-ended products were ligated into Zero Blunt PCR vector (Invitrogen), and the resulting DNA was used to transform TOP10F cells (Invitrogen). Recombinant clones were screened by restriction enzyme digestion using *Eco*RI and *Hin*dIII to confirm the presence of concerted products. In total, 24 clones having the correct size concerted products were sequenced using custom primers (KKPBlunt244: 5′-GGTGACGCGTTAGAATACTCAAGC-3′, and ccd665-R: 5′-GCCCCGGCGTGTCAATAATATC-3′) to analyze the LTR-target junction and host site duplications. The majority of the clones (22 out of 24) had the expected target DNA sequence duplication size of 6-bp. Sequence logos (Supplementary Fig. 9) were generated from 21 unique clones using WebLogo^68^ and show similar integration target sequence preference to those previously reported for HTLV-1 IN^69,70^. Two clones had deletions of 116-bp and 1026-bp, which could be due to multiple integration into a single target DNA.

### Protein-binding analyses

Various fragments of HTLV-1 IN, fused to Sumo (yeast Smt3) on their N-terminus, were injected into a Superdex200 10/300 SEC column either by itself or after being mixed with an equimolar amount of Sumo-fused B56γ(11–380). The column was operated at 4 °C with a flow rate of 0.4 ml min^−1^, and the elution buffer contained 20 mM Tris-HCl (pH 8.0), 0.5 M NaCl, 1 mM MgCl_2_, and 0.5 mM TCEP. In total, 83.5 nanomoles of each protein or complex were brought up to a standardized volume of 242 μL with the running buffer, prior to each sample injection. Protein complex formation was assessed by monitoring the elution profiles and analyzing the collected fractions by SDS-PAGE.

### Reporting summary

Further information on research design is available in the Nature Research Reporting Summary linked to this article.

## Supplementary information

Supplementary Information

Peer Review

Reporting Summary

## Data Availability

Atomic coordinates and the cryo-EM density map have been deposited in the Protein Data Bank and the Electron Microscopy Data Bank (EMDB) under accession code 6VOY and EMD-21301, respectively. All other data are available from the authors upon request.
