# Supplementary material for: Structural basis of host protein hijacking in human T-cell leukemia virus integration
Source: Nat Commun. 2020 Jun 19;11:3121. doi: 10.1038/s41467-020-16963-6 (PMC7305164; doi:10.1038/s41467-020-16963-6)
Supplement: Supplementary file 1 — Supplementary Information [file 41467_2020_16963_MOESM1_ESM.pdf]

Supplementary Information

Structural basis of host protein hijacking in human T-cell leukemia virus integration

Veer Bhatt†, Ke Shi†, Daniel J. Salamango†, Nicholas H. Moeller, Krishan Pandey, Sibes Bera, Heather O. Bohl, Fredy Kurniawan, Kayo Orellana, Wei Zhang, Duane P. Grandgenett, Reuben S. Harris, Anna C. Sundborger-Lunna*, Hideki Aihara*

† Co-first authors
*Correspondence to: asundbor@umn.edu, aihar001@umn.edu

This PDF file includes:

Supplementary Figures 1 to 10
Supplementary Table 1
Supplementary References

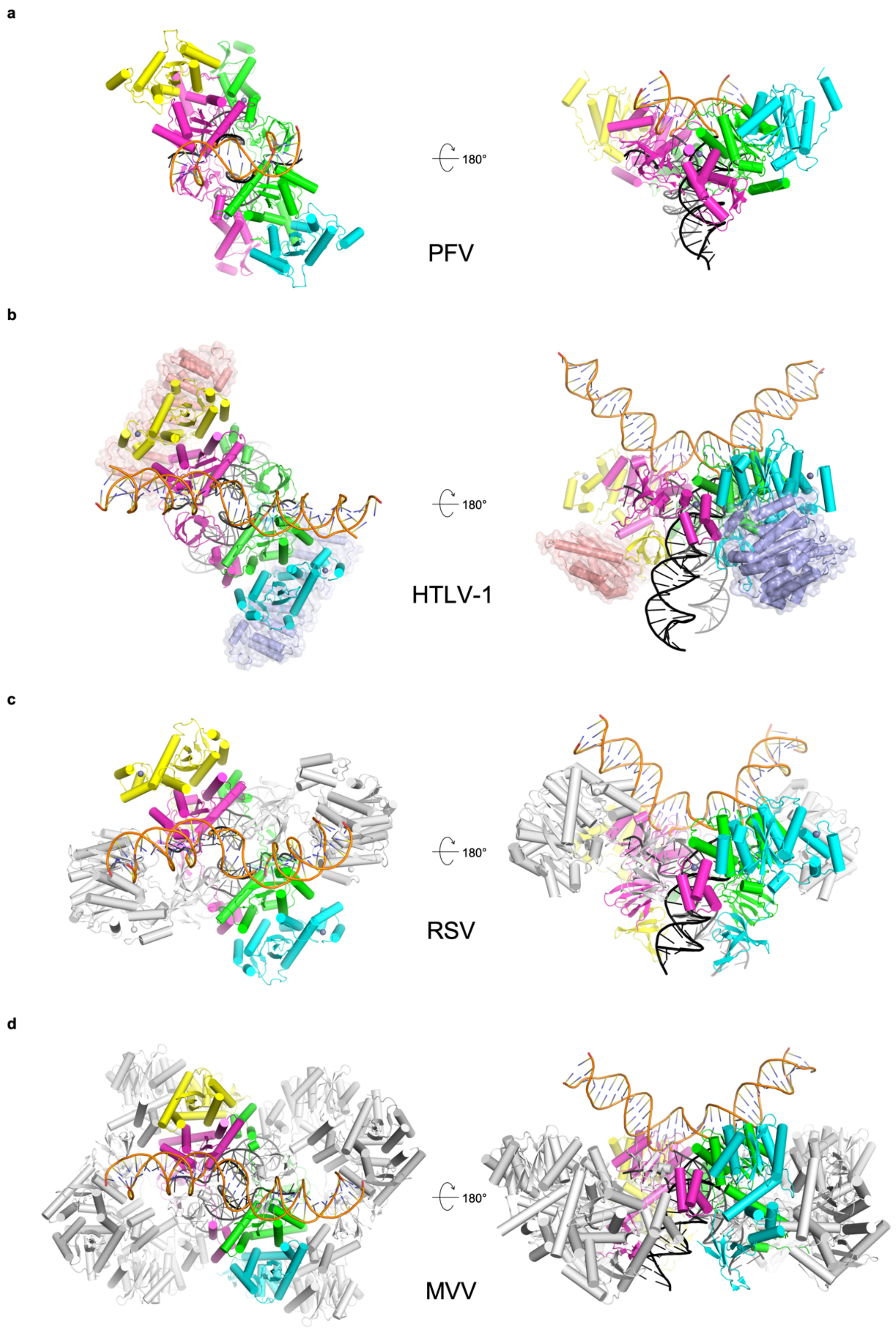

Supplementary Fig. 1 | Diverse retroviral intasome (STC) assemblies. a, Tetrameric prototype foamy virus (PFV) intasome[1]. **b**, Tetrameric HTLV-1 intasome with the host co-factor B56γ (this study). Transparent surface is shown for B56γ. **c**, Octameric Rous sarcoma virus (RSV) intasome[2]. **d**, Hexadecameric maedi-visna virus (MVV) intasome[3]. The core IN tetramers are colored in the same scheme; inner INs in green and magenta, and outer INs in cyan and yellow. Viral and target DNA are shown as black and orange tubes, respectively. Zinc ions are shown as gray spheres.

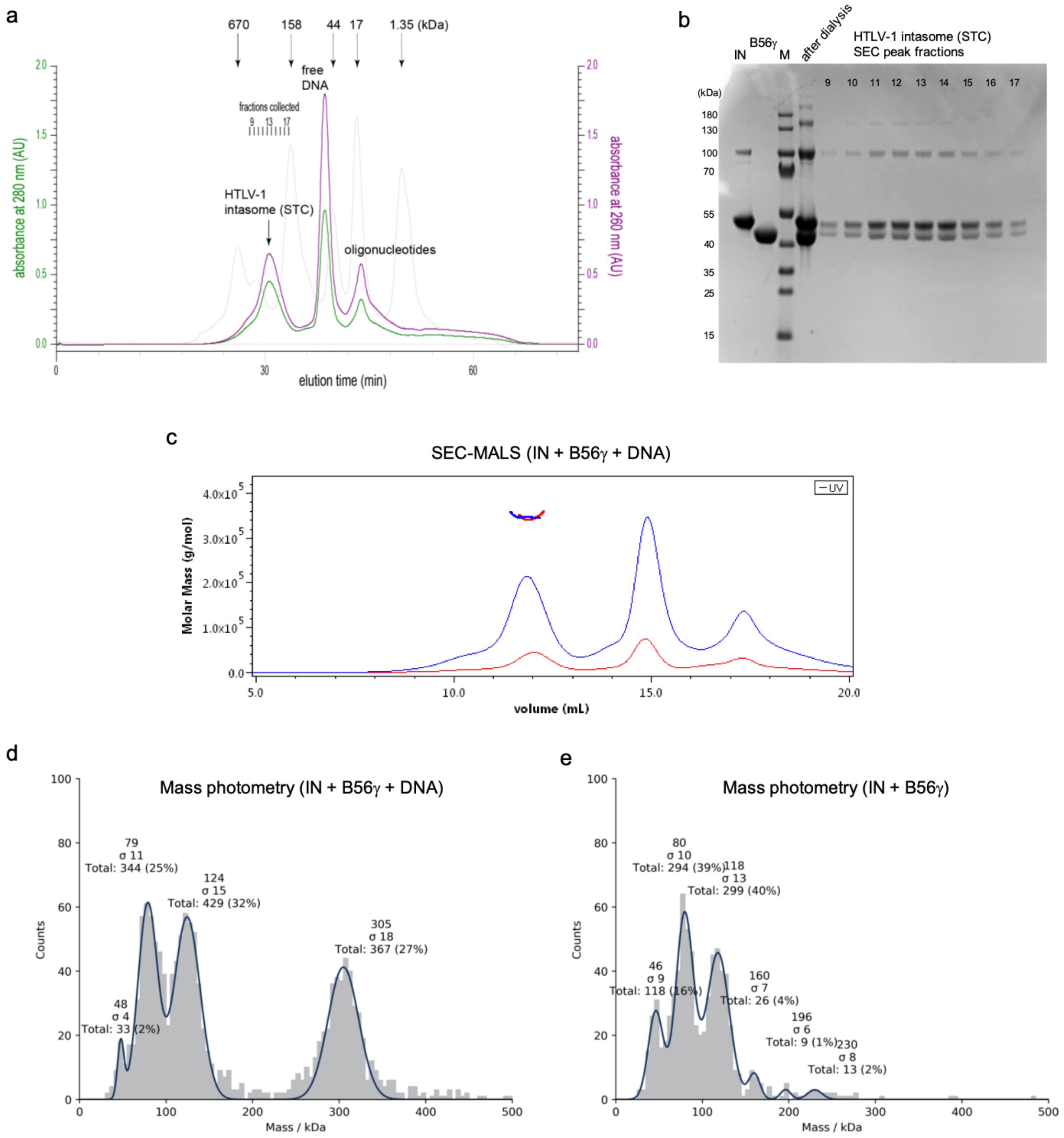

Supplementary Fig. 2 | Biochemical and biophysical characterization of HTLV-1 intasome. a, Isolation of HTLV-1 intasome (strand-transfer complex, STC) by SEC. The chromatogram was obtained by injecting an IN-B56γ-DNA mixture after dialysis into a Superdex200 10/300 column operating at 0.4 mL min^{-1} and detecting UV absorption at 260 nm (purple) and 280 nm (green). The elution profile for molecular mass standards is overlaid, with the size of each protein indicated. Based on re-injections of the isolated complex peak fraction after various incubation time (not shown), the half-life of the isolated complex was estimated to be 1~2 days. **b,** SDS-PAGE analysis of the peak fractions of HTLV-1 intasome (STC) isolated by SEC. **c,** SEC-MALS analysis of the sample similar to that in (**a**), at two different concentrations. The solid lines show UV absorbance and the dots show the weight-average molar mass (Mw) measured every 2 sec across the eluting peak. The sample that generated the blue traces was diluted 5-fold to give the red traces. **d, e,** Mass photometry analyses of the SEC-isolated HTLV-1 intasome (**d**) and an IN-B56γ mixture without DNA (**e**). The histograms show distribution of molecular mass estimates for individual absorption events on the cover slip surface. The HTLV-1 intasome is detected with a median mass of 305 kDa. The ~80 kDa and ~120 kDa peaks are likely to correspond to IN dimer and IN dimer bound to B56γ, respectively. We suspect that the protein-DNA complex is underrepresented in the histogram in (**d**), because of preferential absorption of DNA-free particles on the cover slip surface.

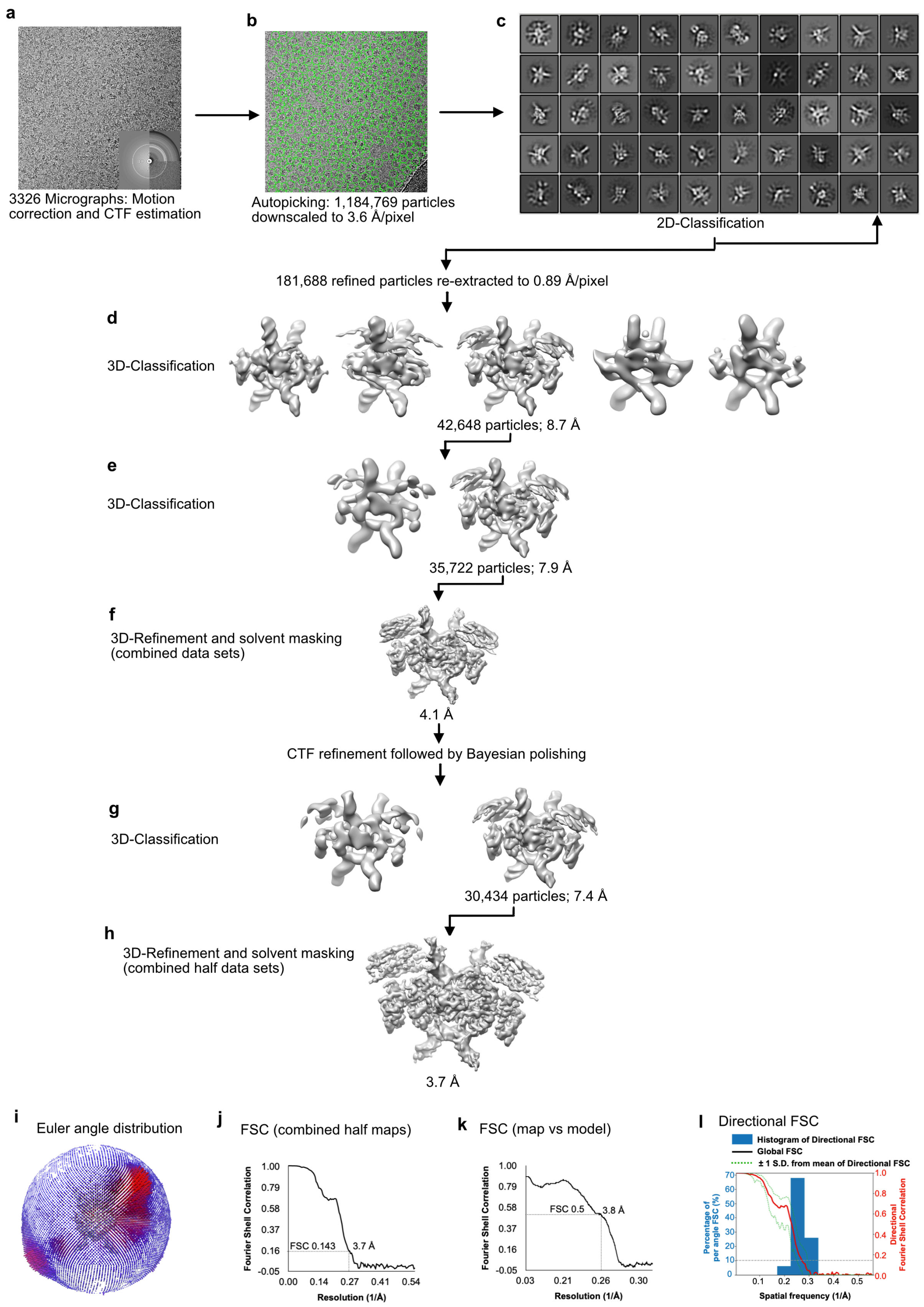

Supplementary Fig. 3 | Cryo-EM data processing workflow. a, A motion corrected micrograph with the result of CTF estimation in lower right corner– the white circle marks 3.4-Å resolution. **b**, Automated particle picking for the same micrograph. **c**, Representative 2D-classes. **d**, **e**, Final classes of 3D-classifications of particles. **f,**

3D refined map resulting after combining the half-maps and solvent flattening. **g**, 3D-classification of polished particles. **h**, The final refined map after combining the two half-data sets and solvent flattening. **i**, Angular distribution of the views. **j**, Half-maps FSC curve. **k**, Map to model FSC curve. **l**, Plot showing the spread of directional FSC curves[4].

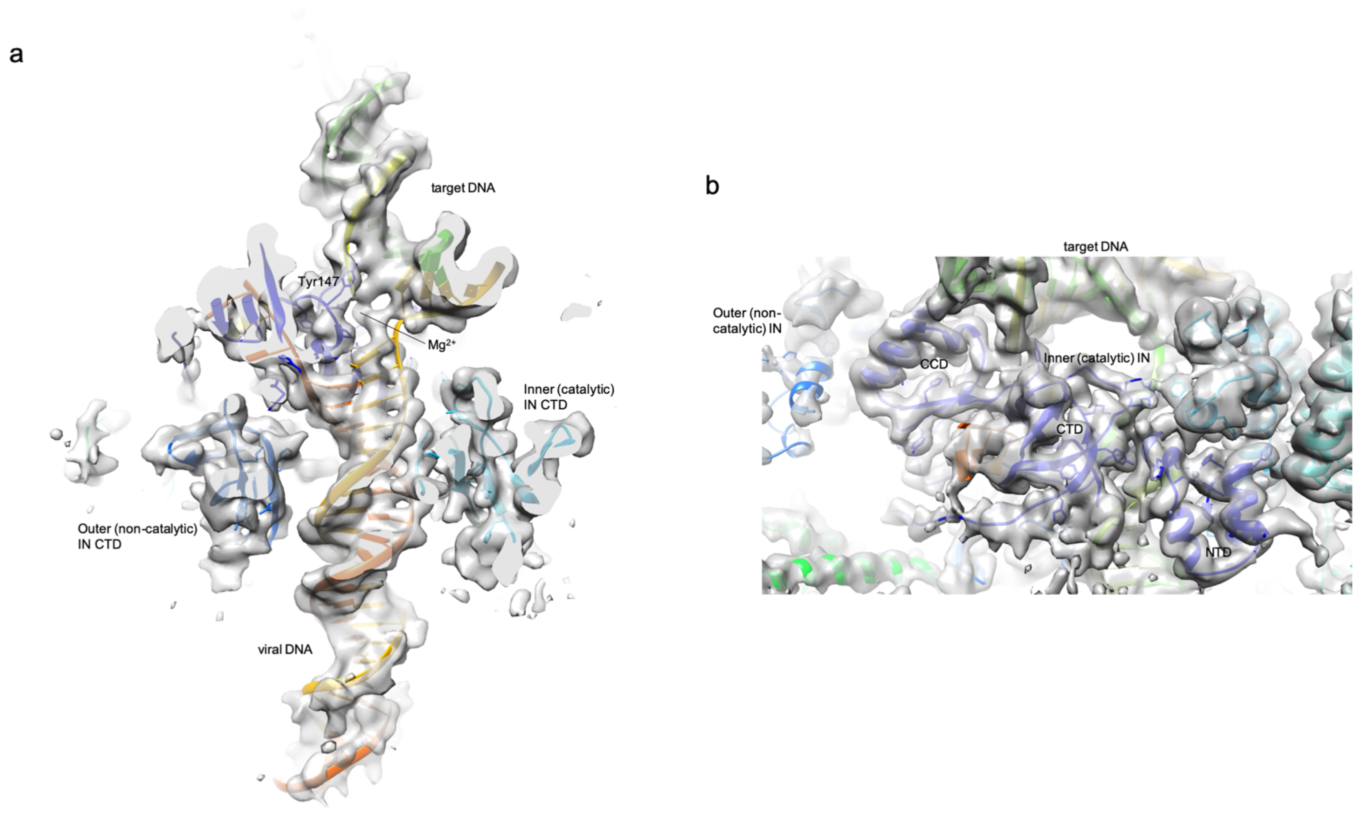

Supplementary Fig. 4 | cryo-EM map for various regions of the complex. a, Viral DNA and the viral/target DNA junction. **b,** Inner catalytic IN protomer complete with 3 domains. The color scheme in this figure differs from that used in all other figures. CCD: catalytic core domain, CTD: C-terminal domain, NTD: N-terminal domain.

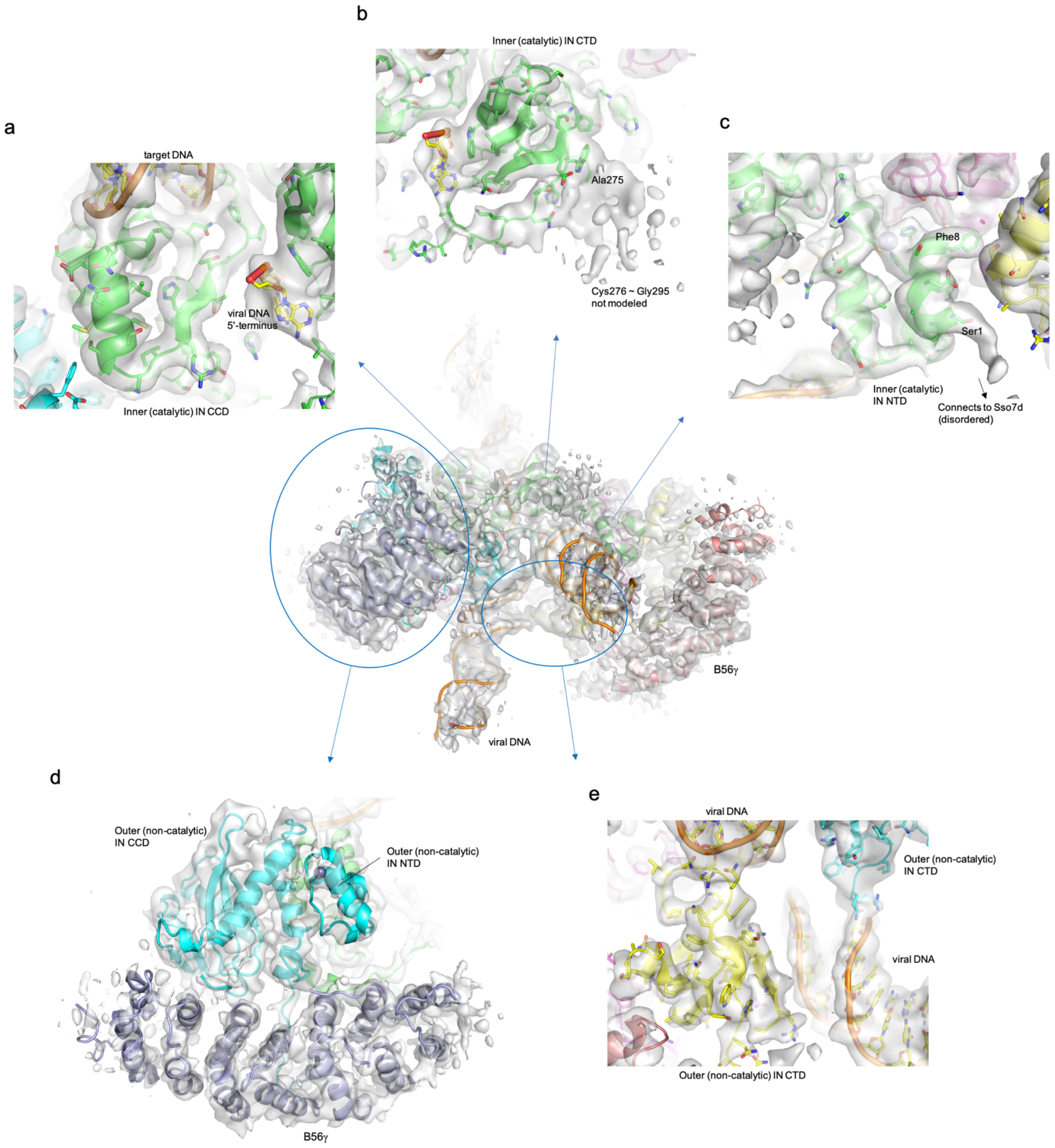

Supplementary Fig. 5 | cryo-EM map for various regions of the complex (continued). a, Inner catalytic IN CCD. **b**, Catalytic IN CTD with an extra density adjacent to the last modeled residue Ala275, likely corresponding to the flexible last 20 residues of HTLV-1 IN. **c**, Catalytic IN NTD with a small extra density adjacent to Ser1 corresponding to the flexible linker that connects IN to Sso7d (not resolved, presumably due to its random positioning). **d**, B56γ and an outer (non-catalytic) IN NTD/CCD. **e**, Outer (non-catalytic) IN CTDs and viral DNAs. A global view of the HTLV-1 intasome is shown in the middle as a reference.

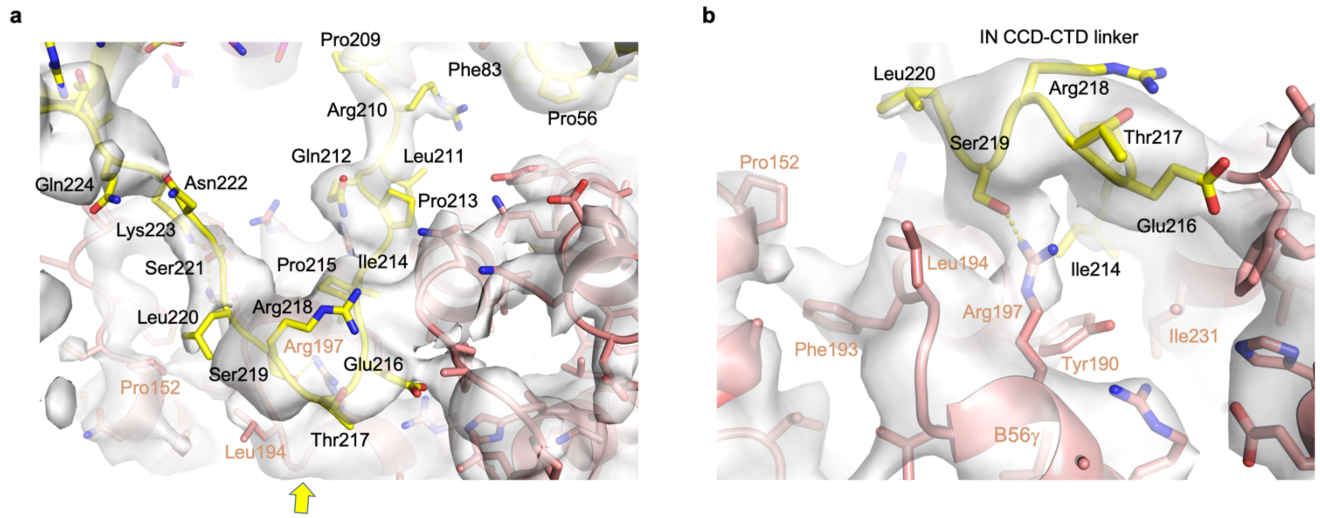

Supplementary Fig. 6 | IN-B56γ interface. a, The outer (non-catalytic) IN CCD-CTD linker fit in the peptide-binding cleft of B56γ. The cryo-EM density map is shown as a transparent surface. The yellow arrow indicates the viewpoint in (**b**). **b,** A close-up view of the interface, showing the insertion of IN Ile214 into a hydrophobic pocket and a hydrogen-bond between IN Ser219 and B56γ Arg197.

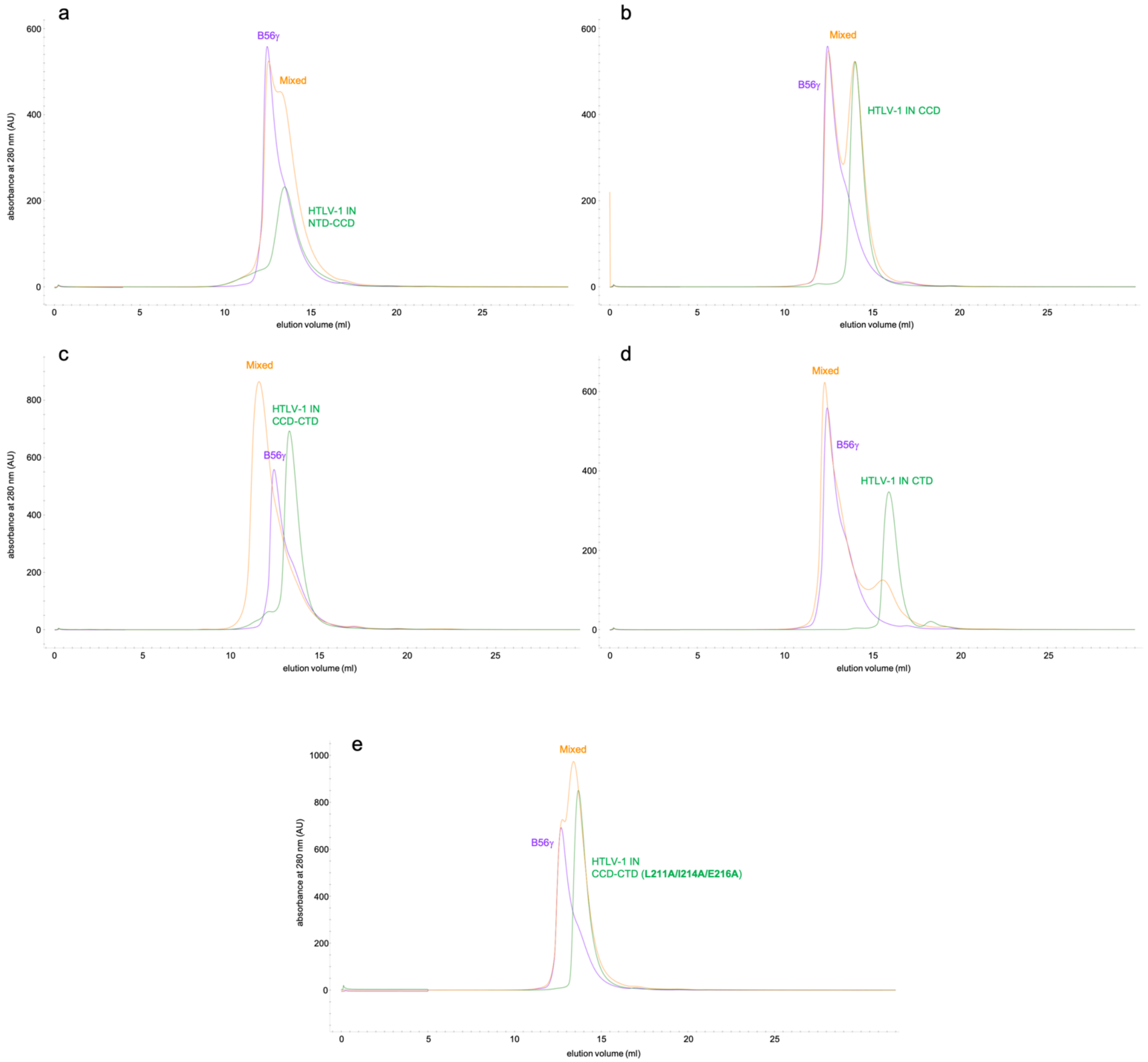

Supplementary Fig. 7 | IN-B56γ binding analysis. Overlay of SEC profiles for IN alone (green), B56γ alone (purple), and 1:1 mixture of the two proteins (orange). **a,** HTLV-1 NTD-CCD showing no association, **b,** HTLV-1 CCD showing no association, **c,** HTLV-1 CCD-CTD showing a stable complex formation, **d,** HTLV-1 CTD showing partial association, **e,** HTLV-1 CCD-CTD (L211A/I214A/E216A) showing no association.

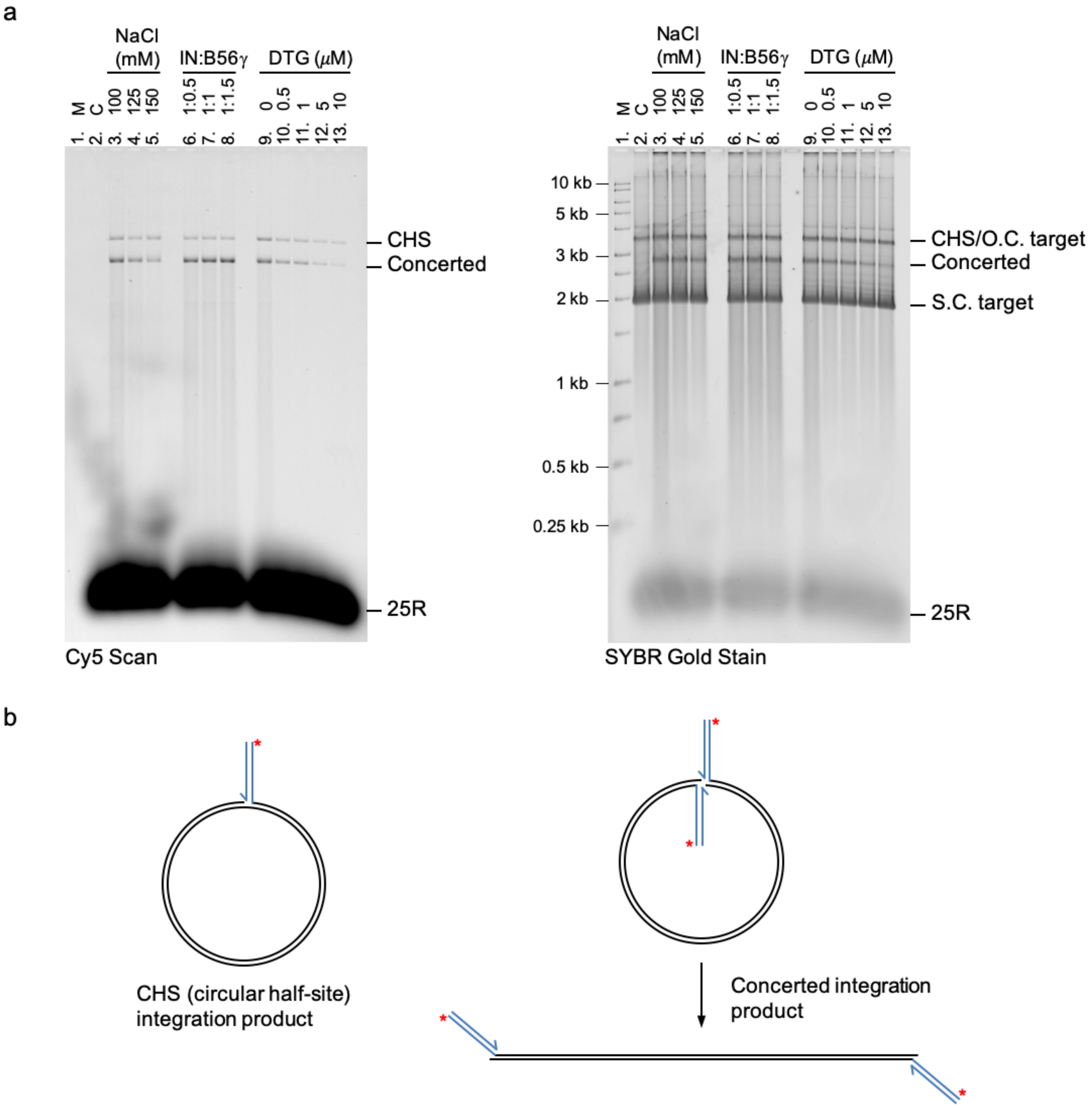

Supplementary Fig. 8 | Characterization of Sso7d-HTLV-1 IN concerted integration activity. a, *In vitro* integration activity of Sso7d(W24A/R43E)-HTLV-1 IN(wt) with Cy5-labeled viral DNA substrate 25R, tested at indicated NaCl concentrations (lanes 3-5). The NaCl concentration of 100 mM was found to be optimal for strand transfer activity. Presence of a cellular co-factor B56γ(11-380) (lanes 6-8) had modest stimulatory effect on concerted integration at IN:B56γ ratio of 1:1.5 (by 24%; lane 3 *vs.* 8). Following preincubation of IN-DNA mixture at 14°C, B56γ was added and further incubated at 14°C for 15 min before addition of target DNA. A titration of dolutegravir (DTG, lanes 9-13) showed dose-dependent inhibition of HTLV-1 strand transfer activities. DTG was added immediately before the addition of target DNA in these reactions. The gel was scanned for Cy5 fluorescence (left), and subsequently stained with SYBR Gold to visualize the target DNA (right). Lane 1, marked "M" contains the MW markers (Promega kb ladder). Lane 2, marked "C" does not contain IN. S.C. target and O.C. target denote supercoiled and open circular target DNA, respectively. **b,** Schematic drawings of the two integration products.

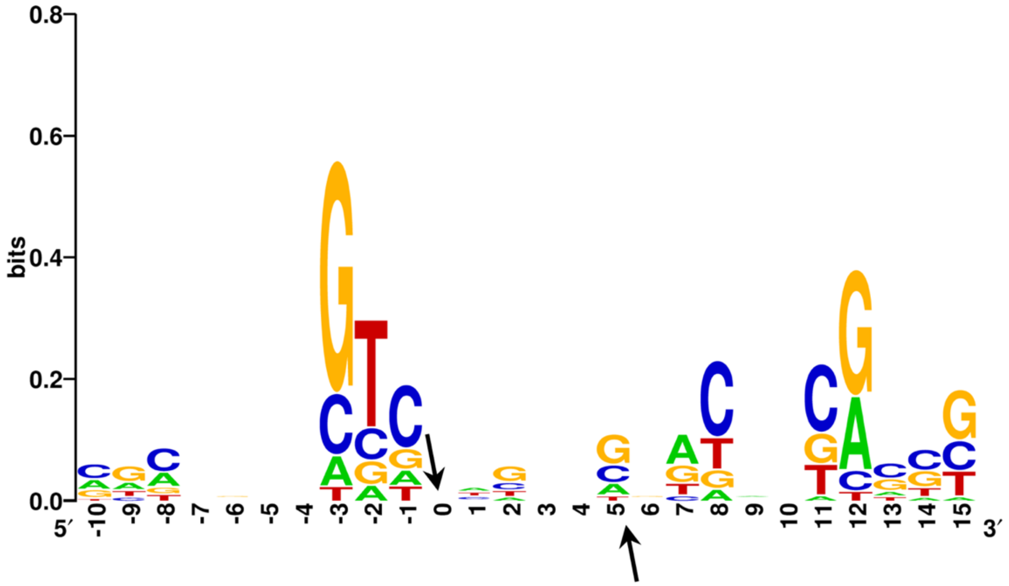

Supplementary Fig. 9 | Characterization of Sso7d-HTLV-1 IN concerted integration products. Sequence logos representing the nucleotide frequency at HTLV-1 integration sites *in vitro*. Arrowheads point to site of strand-transfer. This figure was generated using WebLogo[5].

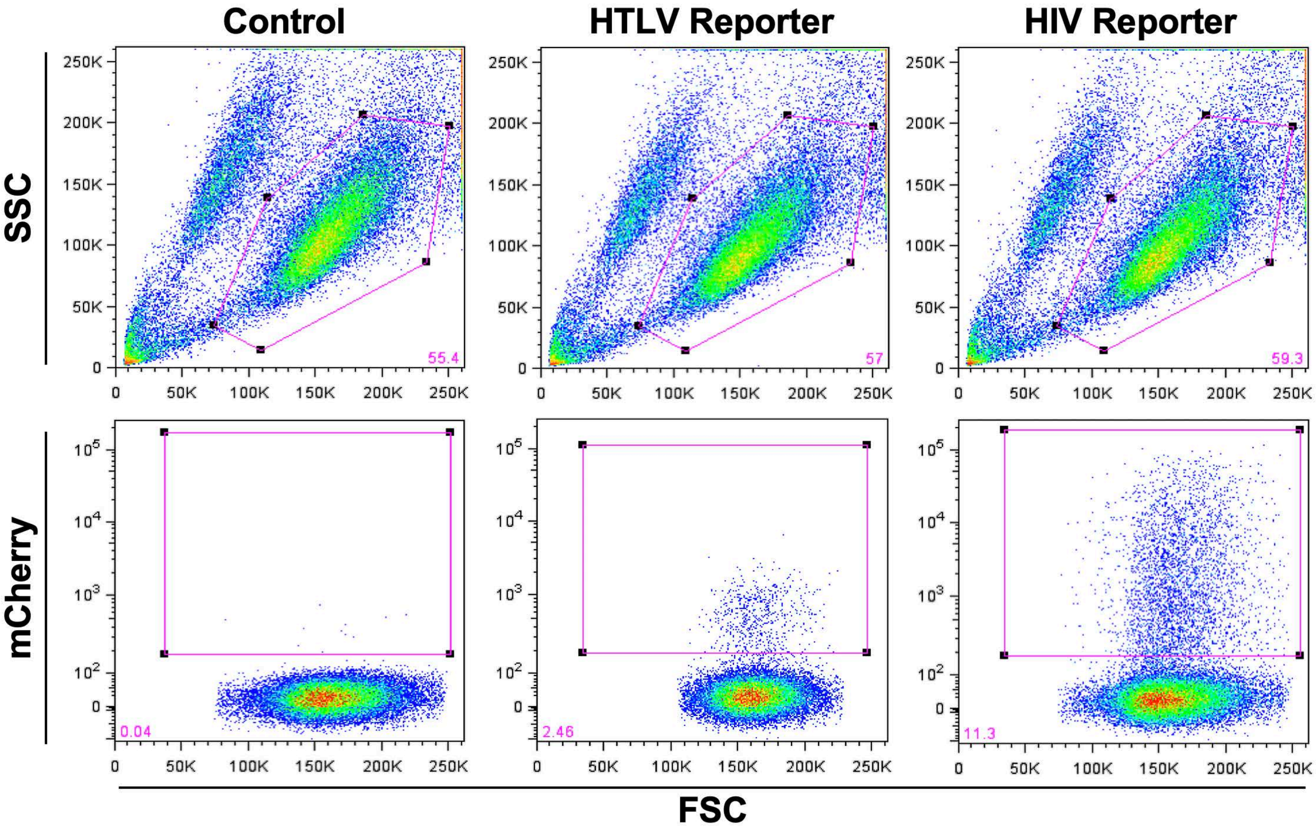

Supplementary Fig. 10 | Flow cytometry example plots. Representative flow cytometry plots of the gating strategy used to determine infection events in control (left), HTLV-1 (middle), and HIV-1 (right) samples expressing the reverse-intron containing reporter vectors.

Supplementary Table 1 | Protein and oligonucleotide sequences used in the structural studies

>Sso7d(W24A/R43E)-HTLV1-IN(wt)
MGSSHHHHHHSSGLVPRGSHMATVKFKYKGEEKEVDISKIKKVARVGKMISFTYDEGGGKTGEGAVSEKDAPKELLQML EKQKKGGSLEVLFQGPSPAELHSFTHCGQTALTLQGATTTEASNILRSCHACRKNNPQHQMPRGHIRRGLLPNHIWQGD ITHFKYKNTLYRLHVWVDTFSGAISATQKRKETSSEAISSLLQAIAYLGKPSYINTDNGPAYISQDFLNMCTSLAIRHT THVPYNPTSSGLVERSNGILKTLLYKYFTDKPDLPMDNALSIALWTINHLNVLTNCHKTRWQLHHSPRLQPIPETRSLS NKQTHWYYFKLPGLNSRQWKGPQEALQEAAGAALIPVSASSAQWIPWRLLKRAACPRPVGGPADPKEKDHQHHG-
>Sso7d(W24A/R43E)-HTLV1-IN(E156Q)
MGSSHHHHHHSSGLVPRGSHMATVKFKYKGEEKEVDISKIKKVARVGKMISFTYDEGGGKTGEGAVSEKDAPKELLQML EKQKKGGSLEVLFQGPSPAELHSFTHCGQTALTLQGATTTEASNILRSCHACRKNNPQHQMPRGHIRRGLLPNHIWQGD ITHFKYKNTLYRLHVWVDTFSGAISATQKRKETSSEAISSLLQAIAYLGKPSYINTDNGPAYISQDFLNMCTSLAIRHT THVPYNPTSSGLVQRSNGILKTLLYKYFTDKPDLPMDNALSIALWTINHLNVLTNCHKTRWQLHHSPRLQPIPETRSLS NKQTHWYYFKLPGLNSRQWKGPQEALQEAAGAALIPVSASSAQWIPWRLLKRAACPRPVGGPADPKEKDHQHHG-
>B56γ(11-380)
MVVDAANSNGPFQPVVLLHIRDVPPADQEKLFIQKLRQCCVLFDFVSDPLSDLKWKEVKRAALSEMVEYITHNRNVITE PIYPEVVHMFAVNMFRTLPPSSNPTGAEFDPEEDEPTLEAAWPHLQLVYEFFLRFLESPDFQPNIAKKYIDQKFVLQLL ELFDSEDPRERDFLKTTLHRIYGKFLGLRAYIRKQINNIFYRFIYETEHHNGIAELLEILGSIINGFALPLKEEHKIFL LKVLLPLHKVKSLSVYHPQLAYCVVQFLEKDSTLTEPVVMALLKYWPKTHSPKEVMFLNELEEILDVIEPSEFVKIMEP LFRQLAKCVSSPHFQVAERALYYWNNEYIMSLISDNAAKILPIMFPSLYRNSKT-
>Sumo-HTLV1-IN_NTD-CCD
MGHHHHHHGSLQDSEVNQEAKPEVKPEVKPETHINLKVSDGSSEIFFKIKKTTPLRRLMEAFAKRQGKEMDSLRFLYDG IRIQADQAPEDLDMEDNDIIEAHREQIGGSPAELHSFTHCGQTALTLQGATTTEASNILRSCHACRKNNPQHQMPRGHI RRGLLPNHIWQGDITHFKYKNTLYRLHVWVDTFSGAISATQKRKETSSEAISSLLQAIAYLGKPSYINTDNGPAYISQD FLNMCTSLAIRHTTHVPYNPTSSGLVERSNGILKTLLYKYFTDKPDLPMDNALSIALWTINHLNVLTNCHKTRWQLHHS P-
>Sumo-HTLV1-IN_CCD
MGHHHHHHGSLQDSEVNQEAKPEVKPEVKPETHINLKVSDGSSEIFFKIKKTTPLRRLMEAFAKRQGKEMDSLRFLYDG IRIQADQAPEDLDMEDNDIIEAHREQIGGRRGLLPNHIWQGDITHFKYKNTLYRLHVWVDTFSGAISATQKRKETSSEA ISSLLQAIAYLGKPSYINTDNGPAYISQDFLNMCTSLAIRHTTHVPYNPTSSGLVERSNGILKTLLYKYFTDKPDLPMD NALSIALWTINHLNVLTNCHKTRWQLHHSP-
>Sumo-HTLV1-IN_CCD-CTD
MGHHHHHHGSLQDSEVNQEAKPEVKPEVKPETHINLKVSDGSSEIFFKIKKTTPLRRLMEAFAKRQGKEMDSLRFLYDG IRIQADQAPEDLDMEDNDIIEAHREQIGGRRGLLPNHIWQGDITHFKYKNTLYRLHVWVDTFSGAISATQKRKETSSEA ISSLLQAIAYLGKPSYINTDNGPAYISQDFLNMCTSLAIRHTTHVPYNPTSSGLVERSNGILKTLLYKYFTDKPDLPMD NALSIALWTINHLNVLTNCHKTRWQLHHSPRLQPIPETRSLSNKQTHWYYFKLPGLNSRQWKGPQEALQEAAGAALIPV SASSAQWIPWRLLKRAACPRPVGGPADPKEKDLQHHG-
>Sumo-HTLV1-IN_CTD
MGHHHHHHGSLQDSEVNQEAKPEVKPEVKPETHINLKVSDGSSEIFFKIKKTTPLRRLMEAFAKRQGKEMDSLRFLYDG IRIQADQAPEDLDMEDNDIIEAHREQIGGSPRLQPIPETRSLSNKQTHWYYFKLPGLNSRQWKGPQEALQEAAGAALIP VSASSAQWIPWRLLKRAACPRPVGGPADPKEKDLQHHG-
>U5-25T20
5' CCAGGAGAGAAATTTAGTACACAGATATCCACCCTAGTCAAGTGTGTCC 3'

>U5-nj25
5' ACTGTGTACTAAATTTCTCTCCTGG 3'
>T20
5′ GGACACACTTGACTAGGGTG 3'
>U5-25c
5' Cy5-CCAGGAGAGAAATTTAGTACACA 3'

Supplementary References:

1 Maertens, G. N., Hare, S. & Cherepanov, P. The mechanism of retroviral integration from X-ray structures of its key intermediates. *Nature* **468**, 326-329, doi:10.1038/nature09517 (2010).

2 Yin, Z. *et al.* Crystal structure of the Rous sarcoma virus intasome. *Nature* **530**, 362-366, doi:10.1038/nature16950 (2016).

3 Ballandras-Colas, A. *et al.* A supramolecular assembly mediates lentiviral DNA integration. *Science* **355**, 93-95, doi:10.1126/science.aah7002 (2017).

4 Tan, Y. Z. *et al.* Addressing preferred specimen orientation in single-particle cryo-EM through tilting. *Nat Methods* **14**, 793-796, doi:10.1038/nmeth.4347 (2017).

5 Crooks, G. E., Hon, G., Chandonia, J. M. & Brenner, S. E. WebLogo: a sequence logo generator. *Genome Res* **14**, 1188-1190, doi:10.1101/gr.849004 (2004).
