# Supplementary material for: Structural basis of host protein hijacking in human T-cell leukemia virus integration
Source: Nat Commun. 2020 Jun 19;11:3121. doi: 10.1038/s41467-020-16963-6 (PMC7305164; doi:10.1038/s41467-020-16963-6)
Supplement: Supplementary file 2 — Peer Review [file 41467_2020_16963_MOESM2_ESM.pdf]

Reviewers' comments:

Reviewer #1 (Remarks to the Author):

This is an exciting and important manuscript describing the characterization of the HTLV-1 IN:DNA intasome, in complex with the B56γ(B΄γ) subunit of an essential host enzyme, protein phosphatase 2A (PP2A), using cryo-electron microscopy. The structure is novel and shows the interactions of two PP2A B56γ molecules in complex with an IN tetramer in the presence of a branched DNA molecule. This interaction is primarily through a loop region localized between the IN CCD and CTD domains, which serves different functions in related retroviruses. Mutagenesis studies and viral titers validate the importance of this inter-domain region.

Specific comments:

1. The manuscript states that the B56γ was "essential for a stable HTLV IN-DNA complex formation". Comment whether any intasome complex in the absence of B56γ was observed on any of the size and MW analysis presented in the manuscript (Extended Fig 2).

2. Results, Extended Data Fig. 2. This is an essential figure to analyze the stoichiometry of the complex. 2A. Molecular standards should be presented in a plot to clearly indicate their exact molecular weights. Consistency between the fraction numbers of Figure 2A, and 2B should be very convincing.

3. Page 7, first paragraph. Comment on whether the presence of B56γ functions on stability of the intasome complex alone or has an effect on target-site preference.

4. Page 14 and extended Fig. 6. The stimulation by B56γ to IN integration is reported as "modest". Was this quantified and how does it compare to previous studies cited (ref. 28)

5. Extended Fig 3. Provide addition information regarding the Sso7D domain. Is it structured? Does it sterically block assembly of potential higher ordered oligomers or B56γ binding?

6. Method of HTLV intasome preparation. After dialyses of intasome assembly mixture against buffer with 100 mM NaCl, the assembly mixture is supplemented with 150 mM NaCl, and incubate for 30 min. Please clarify the purpose of this additional step.

Reviewer #2 (Remarks to the Author):

It was previously shown that serine/threonine protein phosphatase PP2A, including fragment B56γ11-380, interacts with delta-retroviral INs and specifically stimulates HTLV concerted integration (Maertens GN et al. 2016). However, it remained unclear which regions of IN were involved in this interaction and how PP2A-B56γ stimulates HTLV integration. In this work, Bhatt et al. attempt to addresses these questions with the cryo-EM structure of the HTLV1 strand transfer complex intasome bound to the human protein B56γ11-380, as well as biochemical and cell biology experiments. This work provides an important contribution to the field as it shows the first structure of the HTLV intasome and its interaction with the host protein B56γ11-380. These can aid drug development. In addition, this is the first structural report for a Deltaretroviral intasome, which contributes to a better understanding of intasome structural variability. Despite the importance of these findings, the major issue in the manuscript is the confusing conclusions from the biochemical and cell biology experiments. In the absence of further clarification (and some control experiments), the conclusions in the current report are not fully justified.

Major points

The authors state that B56y is essential for the formation of a stable intasome complex, but the in vitro integration assay (Extended Fig. 6) shows that HTLV1 intasomes are apparently equally active in the presence or absence of B56y. This is somewhat confusing. If B56y is essential to form stable intasomes, why does its addition have a negligible effect on integration in vitro? What, if anything, is B56y or PP2A doing in vitro?

Following the same rationale, the cellular data of PP2A / B56y in vivo is generally confusing. Fig. 3 shows an infectivity assay with multiple constructs supporting the role of PP2A-B56y as a specific enhancer for HTLV1 infection. Knockdown of B56y can be interpreted as either (1) affecting viral infectivity or (2) affecting normal cellular function, and thus, indirectly, infectivity. One cannot currently distinguish between these two possibilities. Notably, there is no control to reintroduce B56y back and rescue infectivity/function. This is a key experiment. Similarly, the overexpression data is confusing. If mutant B56g (R197A, or the double mutant L194A/R197A) cannot bind to IN, why would overexpressing it compromise infectivity? Wouldn't one expect overexpression of the mutant to have a minimal effect? Overexpression mutants could also be affecting the host. The overall effect, in cells, appears to behave more like a dominant-negative, affecting the host more than it actually affects the virus.

The authors should clarify how the shRNA-mediated knockdown was conducted. Was the region spanning residues 11-380 on B56y targeted?

It seems to me that the authors were able to obtain a very nice structure, and then wanted to boost the importance of the structural findings with more mechanistic data. But the latter is complicated and confusing, leading to more questions than answers. What is PP2A doing and what is its relevance in HTLV integration? Is it playing an active role in integration? Or is it simply passively hijacked by IN, perhaps in a way that might be similar to HTLV Tax protein.

Minor points

Co-factor typically refers to small molecules. Better to use "accessory factor" to describe PP2A

Shouldn't Extended Data Figure 2 come before Extended Data Figure 1?

Extended Data Figure 5 should have the FL HTLV-1 IN as a positive control. Presumably, it will behave similarly to HTLV-1 CCD-CTD.

Extended Fig. 3 is confusing. I would suggest authors to provide a global view of the intasome and zoom in panels from boxed regions, so that readers can visualize the context of the HTLV intasome with respect to each panels (Ext. Fig. 3c-h).

Local resolution coloring (Fig. 1E-F) can go into Extended Data. The authors also should show a map-to-model FSC to determine the quality of the model fit, and preferably (given that there's fairly strong preferred orientation), a measure of directional resolution (e.g. 3D FSC)

Increase font size for numbers comprising the X and Y axes of plots in Ext. Fig. 2a and Ext. Fig. 5.

Does Sso7d impact oligomerization?

Are the NTD/CTD domains of the outer IN visible

The authors mention on page 4 that HTLV1 resembles RSV intasomes due to the same target site

duplication length comprising 6 bp, although the intasome organization resembles the tetrameric PFV intasome. Since this is the first report of a Deltavirus intasome, it would be useful to have an extended figure showing global differences in protomer organization between intasomes from HTLV-1, PFV, and RSV.

We thank both expert reviewers for their time and constructive comments/suggestions. Our point-by-point responses are shown below in blue.

Reviewers' comments:

Reviewer #1 (Remarks to the Author):

This is an exciting and important manuscript describing the characterization of the HTLV-1 IN:DNA intasome, in complex with the B56γ(B′γ) subunit of an essential host enzyme, protein phosphatase 2A (PP2A), using cryo-electron microscopy. The structure is novel and shows the interactions of two PP2A B56γ molecules in complex with an IN tetramer in the presence of a branched DNA molecule. This interaction is primarily through a loop region localized between the IN CCD and CTD domains, which serves different functions in related retroviruses. Mutagenesis studies and viral titers validate the importance of this inter-domain region.

Specific comments:

1. The manuscript states that the B56γ was "essential for a stable HTLV IN-DNA complex formation". Comment whether any intasome complex in the absence of B56γ was observed on any of the size and MW analysis presented in the manuscript (Extended Fig 2).

In the absence of B56γ we did not observe formation of any HTLV IN-DNA complex, as mentioned in Results section:

"*We found the presence of B56γ to be essential for a stable HTLV-1 IN-DNA complex formation, consistent with…*"

A statement was also added in Method section to clarify this point:

"*No IN-DNA complex was formed in the absence of B56γ(11-380).*"

2. Results, Extended Data Fig. 2. This is an essential figure to analyze the stoichiometry of the complex. 2A. Molecular standards should be presented in a plot to clearly indicate their exact molecular weights. Consistency between the fraction numbers of Figure 2A, and 2B should be very convincing.

Please see the revised **Supplementary Figure 1**. We now show an overlay of the SEC elution profile for MW standard as well as the fraction collection window and fraction numbers (for those analyzed by SDS-PAGE).

We also calculated the apparent molecular mass of the intasome to be 287 kDa based on a calibration curve generated from the elution times of these standard proteins. However, because we already mention the mass values determined by SEC-MALS and mass photometry, which are more direct and reliable methods for mass determination, we prefer not to use this number (287 kDa) in the manuscript.

3. Page 7, first paragraph. Comment on whether the presence of B56γ functions on stability of the intasome complex alone or has an effect on target-site preference.

We thank the reviewer for bringing up this important point. A sentence was added in Discussion section:

"*As previously suggested, PP2A may direct HTLV-1 integration into transcriptionally active regions via its interaction with certain chromatin-associated proteins*[28,52,53]."

4. Page 14 and extended Fig. 6. The stimulation by B56γ to IN integration is reported as "modest". Was this quantified and how does it compare to previous studies cited (ref. 28)

The B56γ mediated stimulation was 24% at IN : B56γ ratio of 1 : 1.5 (lane 3 vs 8). We added this information in the figure legend (now **Supplementary Figure 7**).

This observation is more in line with the studies reported in ref #11 than the same group's earlier observation (ref #28). Please see below our response to the first comment from Reviewer-2.

5. Extended Fig 3. Provide addition information regarding the Sso7D domain. Is it structured? Does it sterically block assembly of potential higher ordered oligomers or B56γ binding?

Sso7d is a highly thermostable protein and therefore it is likely that Sso7d remained folded as a domain. However, none of the Sso7d moieties was resolved in the EM map (density missing) suggesting that they are randomly positioned/oriented. Given also the flexible linker between Sso7d and the IN N-terminus, it seems unlikely that Sso7d affected the oligomeric state of IN or the intasome assembly, although we cannot formally rule out such possibility unless we experimentally analyze the HTLV-1 intasome formed using a Sso7d-free IN. Unfortunately, HTLV-1 IN by itself is highly prone to aggregation/precipitation and we were unable to perform any biochemical studies without the Sso7d tag.

To clarify this point we have added the following to **Supplementary Figure 4** legend:

"***c***, *Catalytic IN NTD with a small extra density adjacent to Ser1 corresponding to the flexible linker that connects IN to Sso7d (not resolved, presumably due to its random positioning).*"

6. Method of HTLV intasome preparation. After dialyses of intasome assembly mixture against buffer with 100 mM NaCl, the assembly mixture is supplemented with 150 mM NaCl, and incubate for 30 min. Please clarify the purpose of this additional step.

This step was to re-solubilize some of the precipitated IN-DNA complex after dialysis. We have added this information in the method description.

"… with 150 mM NaCl and left at room temperature for ~30 min, which helped to re-solubilize some of the precipitated IN-DNA complex."

Reviewer #2 (Remarks to the Author):

It was previously shown that serine/threonine protein phosphatase PP2A, including fragment B56y11-380, interacts with delta-retroviral INs and specifically stimulates HTLV concerted integration (Maertens GN et al. 2016). However, it remained unclear which regions of IN were involved in this interaction and how PP2A-B56y stimulates HTLV integration. In this work, Bhatt et al. attempt to addresses these questions with the cryo-EM structure of the HTLV1 strand transfer complex intasome bound to the human protein B56y11-380, as well as biochemical and cell biology experiments. This work provides an important contribution to the field as it shows the first structure of the HTLV intasome and its interaction with the host protein B56y11-380. These can aid drug development. In addition, this is the first structural report for a Deltaretroviral intasome, which contributes to a better understanding of intasome structural variability. Despite the importance of these findings, the major issue in the manuscript is the confusing conclusions from the biochemical and cell biology experiments. In the absence of further clarification (and some control experiments), the conclusions in the current report are not fully justified.

Major points

The authors state that B56y is essential for the formation of a stable intasome complex, but the in vitro integration assay (Extended Fig. 6) shows that HTLV1 intasomes are apparently equally active in the presence or absence of B56y. This is somewhat confusing. If B56y is essential to form stable intasomes, why does its addition have a negligible effect on integration in vitro? What, if anything, is B56y or PP2A doing in vitro?

It was shown previously (Ref #28) that *in vitro* integration activity of HTLV-1 IN is highly sensitive to salt ([NaCl] needs to be as low as 50 mM) and is greatly stimulated by B56γ, in a HEPES-based buffer condition. The same group later showed that this activity is more robust and salt-resistant ([NaCl] up to 200 mM well tolerated) and no longer requires (is only slightly stimulated by) B56γ in a more optimized PIPES-based buffer condition (Ref #11). Our highly soluble Sso7d(W24A/R43E)-fused HTLV-1 IN turned out to be quite active by itself in HEPES buffer at 100 mM, which is stimulated only modestly by B56γ (24% at IN:B56γ ratio of 1:1.5 (lane 3 vs. 8 in **Supplementary Figure 7**), but interestingly, it is much less active in PIPES buffer (data not shown). We also observed that, in a more stringent condition containing 125 mM NaCl, the concerted integration activity of Sso7d(W24A/R43E)-HTLV-1 IN was lower but stimulated by ~70% at IN:B56γ ratio of 1:0.5 or 1:1 (not shown). Thus, the biochemical activities of HTLV IN are highly dependent on the buffer condition and protein construct used. On the other hand, HTLV-1 IN-DNA complex formation *in vitro* strictly required B56γ, regardless of buffer condition. These observations were confusing indeed, and led us to conclude that we need to rely on a cell-based assay to determine whether the stabilization by B56γ is required for HTLV-1 integration in a more biologically meaningful condition.

Of note, this situation parallels that concerning HIV-1 IN and its well-established host co-factor LEDGF/p75. Modest stimulation of the concerted integration activity of HIV-1 IN *in vitro* by

LEDGF or its IBD (integrase binding domain) has been observed (*e.g.* Pandey et al., *J. Virol* 2007, Ref. #46), where higher LEDGF to IN ratios actually inhibited concerted integration. The highly soluble Sso7d-fused HIV-1 IN was hyper-active and exhibited robust concerted integration activity *in vitro* in the absence of LEDGF or IBD. However, when incorporated into virions, it was functional but not hyper-active (Li et al., 2014, PMID: 25119883).

Following the same rationale, the cellular data of PP2A / B56y in vivo is generally confusing. Fig. 3 shows an infectivity assay with multiple constructs supporting the role of PP2A-B56y as a specific enhancer for HTLV1 infection. Knockdown of B56y can be interpreted as either (1) affecting viral infectivity or (2) affecting normal cellular function, and thus, indirectly, infectivity. One cannot currently distinguish between these two possibilities. Notably, there is no control to reintroduce B56y back and rescue infectivity/function. This is a key experiment. Similarly, the overexpression data is confusing. If mutant B56g (R197A, or the double mutant L194A/R197A) cannot bind to IN, why would overexpressing it compromise infectivity? Wouldn't one expect overexpression of the mutant to have a minimal effect? Overexpression mutants could also be affecting the host. The overall effect, in cells, appears to behave more like a dominant-negative, affecting the host more than it actually affects the virus.

We appreciate that interpretation of the results of these experiments can be complicated by potential off-target effect of shRNA knockdown or the indirect effect of B56γ knockdown on virus infectivity. To mitigate these possible problems we performed several complementary experiments and obtained data summarized below.

- Knockdown of nuclear localized B56γ, but not the highly similar cytoplasmic isoform B56α, attenuates integration of HTLV-1. In contrast, HIV-1 integration was not significantly affected by the knockdown of either B56γ or B56α. Of note, efficient and selective depletion of the targeted transcript in these shRNA-expressing cell lines was confirmed previously in our publication, and it was shown that neither B56γ nor B56α knockdown has discernable effect on the cell cycle (Ref #39).
- Overexpression of B56γ variants with specific mutations that compromise IN-binding attenuates integration of HTLV-1 but not that of HIV-1.
- Inhibiting all isoforms of B56 by the peptide-based PP2A-B56 inhibitor (developed and characterized in Ref #40) attenuates integration of HTLV-1 to a similar level to those observed in the above two experiments, but had minimal effect on HIV-1 integration.

We tested HTLV-1 and HIV-1 in parallel in all cases, specifically to address the point raised by the reviewer – A defect in general cellular function would have also affected the HIV-1 infectivity. We also used 3 orthogonal approaches: shRNA-knockdown, overexpression of mutant proteins, and expression of a competitive inhibitor, to ensure that the effect we observed indeed concerns B56γ.

As for the dominant-negative effect of overexpressing the R197A mutant, we can think of two scenarios. The first is that B56γ-R197A is still partially interacting with IN (although complex is not stable enough to co-elute in SEC) and therefore the overexpression of this defective protein interferes with intasome formation. Alternatively, HTLV-1 integration in cells may actually

require the PP2A holoenzyme containing B56γ, and overexpressing the defective B56γ depleted the pool of PP2A holoenzyme containing the wild type B56γ capable of supporting IN function. We have added brief description of these possibilities in the manuscript.

The suggested control experiment to add back B56γ to rescue infectivity/function in the shRNA knockdown background would further address the potential off-target effect. The experiment is tricky as our shRNA construct targets the coding sequence of B56γ, but it may be possible to generate shRNA-resistant B56γ gene by introducing several synonymous mutations. Unfortunately, due to the COVID-19 pandemic and laboratory lockdown, such experiments will not be possible for the foreseeable future. We believe that our current data, as summarized above, already strongly support the specific involvement of PP2A-B56γ in HTLV-1 integration.

The authors should clarify how the shRNA-mediated knockdown was conducted. Was the region spanning residues 11-380 on B56y targeted?

The shRNA target is around the amino acid residues 91-93, so it is between 11-380. Details of the shRNA knockdown constructs and cell lines are described in REF #39.

It seems to me that the authors were able to obtain a very nice structure, and then wanted to boost the importance of the structural findings with more mechanistic data. But the latter is complicated and confusing, leading to more questions than answers. What is PP2A doing and what is its relevance in HTLV integration? Is it playing an active role in integration? Or is it simply passively hijacked by IN, perhaps in a way that might be similar to HTLV Tax protein.

As discussed above, we believe that our cell-based HTLV-1 *vs*. HIV-1 infectivity data strongly support our structural observation that PP2A-B56γ is repurposed as a critical scaffolding component of the HTLV-1 intasome.

Minor points

Co-factor typically refers to small molecules. Better to use "accessory factor" to describe PP2A

The term 'co-factor' is indeed used to refer to small molecules like vitamins. However, host co-factor (and restriction factor) is also a common term in virology in discussing host-pathogen interaction (*e.g.* HIV-1 IN binds a cellular co-factor LEDGF). In addition, "accessary" implies non-essential. Thus, we would like to keep this term as is.

Shouldn't Extended Data Figure 2 come before Extended Data Figure 1?

In the original submission, these figures were in the order they were cited in the manuscript. However, we agree that it would be more logical to discuss complex formation/characterization before structure determination. Thus, we rearranged these Supplementary figures as suggested.

Extended Data Figure 5 should have the FL HTLV-1 IN as a positive control. Presumably, it will behave similarly to HTLV-1 CCD-CTD.

As the CCD-CTD two-domain fragment is sufficient for stable complex formation, and Sumo-fused FL HTLV-1 IN had a solubility issue, we did not include full-length (FL) IN in this experiment. However, the mass photometry data shown in **Supplementary Fig. 1d, e** (and the cryo-EM structure itself) confirm that the full-length HTLV-1 IN forms a stable complex with B56γ, and we agree with the reviewer that it would have behaved very similarly to CCD-CTD.

Extended Fig. 3 is confusing. I would suggest authors to provide a global view of the intasome and zoom in panels from boxed regions, so that readers can visualize the context of the HTLV intasome with respect to each panels (Ext. Fig. 3c-h).

We thank the reviewer for this excellent suggestion. We have modified this figure (now **Supplementary Figure 4**) as suggested, with a global view in the middle to indicate which region each panel corresponds to.

Local resolution coloring (Fig. 1E-F) can go into Extended Data. The authors also should show a map-to-model FSC to determine the quality of the model fit, and preferably (given that there's fairly strong preferred orientation), a measure of directional resolution (e.g. 3D FSC)

We thank the reviewer for this expert suggestion. We have added map-to-model FSC as well as directional FSC plots in the new **Supplementary Figure 2**.

We agree that the local resolution coloring could be supplementary data. Should the space constraint become an issue we will move these panels to the supplement.

Increase font size for numbers comprising the X and Y axes of plots in Ext. Fig. 2a and Ext. Fig. 5.

We have increased font sizes of the axis labels for these plots to make them readable (now **Supplementary Figures 1 and 6**)

Does Sso7d impact oligomerization?

HTLV-1 IN without Sso7d is prone to aggregation/precipitation and therefore it is difficult to make a direct comparison, but given that Sso7d is monomeric by itself and is tethered to the N-terminus of IN via a disordered linker (e.g. **Supplementary Figure 4c**), it is unlikely to have affected IN oligomerization beyond preventing its non-specific aggregation.

Are the NTD/CTD domains of the outer IN visible

All 3 domains of both the inner and outer IN protomers are visible. To make this point clearer, we have added more labels to indicate the positions of IN structural domains in **Fig. 1**.

The authors mention on page 4 that HTLV1 resembles RSV intasomes due to the same target site duplication length comprising 6 bp, although the intasome organization resembles the tetrameric PFV intasome. Since this is the first report of a Deltavirus intasome, it would be useful to have an extended figure showing global differences in protomer organization between intasomes from HTLV-1, PFV, and RSV.

We thank the reviewer for this excellent suggestion. We have added a new figure (**Supplementary Figure 9**) showing a comparison of PFV (tetrameric), HTLV (tetrameric, with a host factor), RSV (octameric), and MVV (hexadecameric) intasomes representing each type.

REVIEWERS' COMMENTS:

Reviewer #1 (Remarks to the Author):

This is a revised manuscript describing the cryo-EM structure of the HTLV-1 IN:DNA intasome bound to the B56γ subunit of the host PP2A protein. This structure is significant and show the interaction of two PP2A B56γ subunits bound to the IN tetramer in the presence of a branched DNA substrate. The novel feature of the structure is that the interaction is through a loop region between the IN CCD and CTD domains, which has previously not been identified to function in other IN:host factor interactions. The authors have responded to a majority of the previous comments.

These two points remain:
1) Order of supplemental Figures should remain in order of citing (line 54) in the text.
3) Line 76. Change the text regarding the "strong stimulatory effect on deltaretroviral concerted integration reactions".

The authors in their response dedicated an entire paragraph indicating that "the biochemical activities of HTLV IN are highly dependent on the buffer condition and protein construct used." Simply citing this manuscript ignores the entire rebuttal presented by the authors.

Reviewer #2 (Remarks to the Author):

The structure of the HTLV1 intasome is an important contribution to the scientific community and the determination of its structure bound to the cellular factor B56y is a plus. However, the inconsistence in the biology does not allow authors to confidently claim that B56y is required for intasome assembly and integration of the HTLV1 genome into the host DNA. In vitro experiments suggest B56y is essential for intasome assembly, but both in vitro and in vivo assays show integration of HTLV1 in absence of B56y. Is B56y essential or an enhancer for HTLV1 cell infection? The impact on infectivity by HTLV1 mutants (L194A/R197A) that should have either none or minor interaction with virus integrase also leave important open questions.

We thank both expert reviewers for their time and further comments/suggestions. Our point-by-point responses are shown below in blue.

REVIEWERS' COMMENTS:

Reviewer #1 (Remarks to the Author):

This is a revised manuscript describing the cryo-EM structure of the HTLV-1 IN:DNA intasome bound to the B56γ subunit of the host PP2A protein. This structure is significant and show the interaction of two PP2A B56γ subunits bound to the IN tetramer in the presence of a branched DNA substrate. The novel feature of the structure is that the interaction is through a loop region between the IN CCD and CTD domains, which has previously not been identified to function in other IN:host factor interactions. The authors have responded to a majority of the previous comments.

These two points remain:
1) Order of supplemental Figures should remain in order of citing (line 54) in the text.

The supplementary figures have been rearranged so that they are cited in order.

3) Line 76. Change the text regarding the "strong stimulatory effect on deltaretroviral concerted integration reactions".

The authors in their response dedicated an entire paragraph indicating that "the biochemical activities of HTLV IN are highly dependent on the buffer condition and protein construct used." Simply citing this manuscript ignores the entire rebuttal presented by the authors.

We have added a phrase "under certain biochemical conditions" and another reference (#11, which shows a relaxed requirement for B56γ in deltaretroviral integration) to this sentence.

Reviewer #2 (Remarks to the Author):

The structure of the HTLV1 intasome is an important contribution to the scientific community and the determination of its structure bound to the cellular factor B56y is a plus. However, the inconsistence in the biology does not allow authors to confidently claim that B56y is required for intasome assembly and integration of the HTLV1 genome into the host DNA. In vitro experiments suggest B56y is essential for intasome assembly, but both in vitro and in vivo assays show integration of HTLV1 in absence of B56y. Is B56y essential or an enhancer for HTLV1 cell infection? The impact on infectivity by HTLV1 mutants (L194A/R197A) that should have either none or minor interaction with virus integrase also leave important open questions.

We have done the following rewording to soften our statements regarding the requirement for B56γ.

- In Abstract: "Our studies reveal a ~~critical~~ ***key*** host-virus interaction underlying the replication of an important human pathogen…"

- Page 3, at the end of INTRODUCTION: "PP2A-B56γ is an integral component of the HTLV-1 intasome and it plays ~~a critical~~ ***an important*** role in HTLV-1 infection."
- Page 7, at the end of RESULTS: "Taken together, these results support our structural data that B56γ is ~~a critical~~ ***an important*** component of the HTLV-1 intasome."
- Figure 3 title: B56γ ~~is required for~~ ***plays an important role in*** HTLV-1 infection.

Possible explanations for the dominant and negative effect of overexpressing the mutant variants of B56γ on HTLV-1 integration had been provided in our previous rebuttal and the revised manuscript.
